# Serum uric acid reduction through SGLT2 inhibitors: evidence from a systematic review and meta-analysis

**DOI:** 10.3389/fphar.2025.1551390

**Published:** 2025-06-19

**Authors:** Shiwen Yang, Qiaozhi Hu, Kexin Liu, Binjie Xiao, Bofei Zhang, Na Su

**Affiliations:** ^1^ Department of Pharmacy, West China Hospital, Sichuan University, Chengdu, China; ^2^ Department of Pharmacy, Jiangxi Mental Health Center, Nanchang, China; ^3^ State Key Laboratory of Quality Research in Chinese Medicine, Institute of Chinese Medical Sciences, University of Macau, Macau, Macao SAR, China; ^4^ West China School of Pharmacy, Sichuan University, Chengdu, China

**Keywords:** sodium-glucose cotransporter-2 inhibitor, with and without T2DM, serum uric acid, gout, meta-analysis

## Abstract

**Background:**

Elevated serum uric acid (SUA) is strongly associated with adverse clinical outcomes. Sodium-glucose-cotransporter-2 (SGLT2) inhibitors not only lower blood glucose levels but also reduce UA. However, comparative data on the SUA-lowering effects among different SGLT2 inhibitors remain sparse, hindering evidence-based drug selection. This study aimed to systematically evaluate the effects of various SGLT2 inhibitors on SUA.

**Methods:**

We searched the Cochrane Central Register of Controlled Trials (Ovid SP), Embase (Ovid SP), PubMed, and ClinicalTrials.gov up to March 2024 for randomized controlled trials (RCTs) evaluating SGLT2 inhibitors in patients with or without type 2 diabetes mellitus (T2DM). The primary outcome was the change in SUA levels compared with placebo. Data were analyzed using Review Manager 5.4. Pooled mean differences (MDs) for continuous outcomes (SUA change) and relative risk (RR) for dichotomous outcomes (gout incidence) were calculated. Study quality was evaluated using the Cochrane Risk of Bias tool (RoB 2), and the overall evidence quality was evaluated using the GRADE approach.

**Results:**

A total of 51 RCTs were included in the meta-analysis. The SUA levels were significantly lower in all SGLT2 inhibitors groups than in the placebo groups. SGLT2 inhibitors have superior efficacy in lowering SUA levels compared with placebo [MD = −32.14 μmol/L, 95% CI (−35.96 to −28.31); *P* < 0.001]. Subgroup analysis showed empagliflozin achieved the greatest reduction in SUA [MD = −45.61 μmol/L, 95% CI (−52.26 to −38.97); *P* < 0.00001], while sotagliflozin had the least effect [MD = −13.72 μmol/L, 95% CI (−19.16 to −8.29); *P* < 0.00001]. The GRADE profiles indicated low-quality evidence for reduction in SUA levels. However, there was no difference in the incidence of gout between the two groups [RR = 0.96, 95% CI (0.77–1.21), *P* = 0.75].

**Conclusion:**

SGLT2 inhibitors demonstrated greater SUA reduction than placebo, highlighting their potential as multifactorial therapies in high-risk populations.

**Systematic Review Registration:**

https://www.crd.york.ac.uk/prospero/#loginpage, identifier CRD42023458993.

## 1 Introduction

Serum uric acid (SUA), the end product of purine metabolism, is primarily excreted through the kidney and digestive tract. Disruptions in SUA metabolism can result in elevated blood levels (hyperuricemia), a major risk factor for cardiovascular, kidney, and metabolic diseases (Li B. et al., 2023; Yuan et al., 2024). For example, elevated SUA levels are commonly detected in patients with type 2 diabetes mellitus (T2DM) (Ito et al., 2011; Katsiki et al., 2013; Kodama et al., 2009), and elevated SUA levels in the general population are associated with an increased risk of developing new-onset diabetes (Lv et al., 2013). Elevated SUA levels have been demonstrated to significantly increase the risk of various metabolic complications, including stroke (Jiang et al., 2025), diabetic retinopathy (Rivera-De-la-Par et al., 2024; Li et al., 2025), diabetic peripheral neuropathy (Fayazi et al., 2022), peripheral arterial disease (Tseng, 2004) and chronic kidney disease (CKD) (Pan et al., 2023). Epidemiological studies have shown that the prevalence of cardiovascular-kidney-metabolic diseases (CKM) in patients with gout is at least twice that observed in individuals without gout (Zhu et al., 2012). Therefore, early intervention and effective management of SUA levels are important in high-risk populations, aiming to reduce SUA to prevent or mitigate the development of associated metabolic complications. However, RCTs in non-gout populations have failed to demonstrate any clear CKM benefit from standard urate-lowering therapy (Doherty et al., 2018; Mackenzie et al., 2020; Badve et al., 2020; Doria et al., 2020). Even febuxostat, a first-line urate-lowering agent, has been issued an FDA issued warning for cardiovascular mortality risk (U.S. FOOD and DRUG ADMINISTRATION, 2019). This highlights the need to find therapeutic agents that not only lower SUA levels effectively but also provide cardiovascular and renal protection. Recently new methodologies in total metabolic management have emerged leveraging stem cell therapy (with physiologic therapies) in diabetes and its complications (Saha et al., 2023). This also suggests that when exploring new uric acid-lowering treatment options, we should holistically consider their potential benefits to enhance overall metabolic health.

Sodium‒glucose cotransporter-2 (SGLT2) inhibitors are a new type of antidiabetic agent that blocks glucose reabsorption in the proximal renal tubules. They increase the amount of glucose removed through the urine and lower serum glucose levels (U.S. FOOD and DRUG ADMINISTRATION, 2021; Scheen, 2015). In addition to their potent hypoglycemic effects, SGLT2 inhibitors confer multiple metabolic benefits, including antihypertensive properties (Chilton et al., 2017), weight reduction (Sargeant et al., 2019), and enhanced cardiovascular and renal protection (Zelniker et al., 2019; Mentz et al., 2023; McDonagh et al., 2023; Lv et al., 2023). With growing evidence supporting their benefits, SGLT2 inhibitors are now used not only in the treatment of T2DM but have also been integrated into several clinical guidelines for treating cardiovascular diseases—particularly heart failure—and CKD (Group KDIGOKCW, 2024; Author Anonymous, 2024; Heidenreich et al., 2022). Notably, evidence indicates that SGLT2 inhibitors also exert a UA-lowering effect (Zhao et al., 2018; Ferrannini et al., 2013). This effect may stem from several mechanisms: promoting UA excretion through diuretic effects (Chino et al., 2014; Lytvyn et al., 2015), regulating renal transporters to reduce UA reabsorption (Vallon, 2024; Dong et al., 2023), and inhibiting purine synthesis via the pentose phosphate pathway while enhancing UA elimination (Packer, 2024). The role of SGLT2 inhibitors in regulating SUA levels is critically important for reducing the incidence of metabolic disorder-related diseases (Packer, 2024). However, it remains unclear whether this reduction is significant when evaluated systematically, whether these effects are consistent across different SGLT2 inhibitors, and whether this effect is relevant in patients without T2DM. Thus, this study aimed to evaluate and compare the effects of different SGLT2 inhibitors on SUA levels in patients with and without T2DM through a systematic review and meta-analysis, and to provide comprehensive evidence for related studies.

## 2 Methods

This systematic review and meta-analysis was performed in accordance with the guidelines established by the Preferred Reporting Items for Systematic Reviews and Meta-Analyses (PRISMA) (Shamseer et al., 2015). This review was registered in PROSPERO, and the registration number is CRD42023458993.

### 2.1 Literature search

We conducted a systematic literature search in the Cochrane Central Register of Controlled Trials (via Ovid SP), Embase (via Ovid SP), PubMed, Web of Science, and Clinical Trials, from database inception to March 2024. The resulting literature was evaluated for eligibility, and the relevant studies were included in the review. Search terms included medical subject headings and keywords related to “Sodium-Glucose Cotransporter-2 Inhibitors”, “SGLT2 inhibitors”, “Canagliflozin”, “Dapagliflozin”, “Empagliflozin”, “Ipragliflozin”, “Luseogliflozin”, “Sotagliflozin”, “Sergliflozin”, “Remogliflozin”, “Tofogliflozin”, “Bexagliflozin”, “Type 2 diabetes” and “randomized controlled trial”.

### 2.2 Study selection

Studies were selected based on the following criteria (Li B. et al., 2023): Study type: publicly published randomized controlled trials (RCTs) limited to the English language (Yuan et al., 2024); Population: patients undergoing treatment with any kind of SGLT2 inhibitor regardless of their underlying disease (Ito et al., 2011); Intervention and Comparator: SGLT2 inhibitors versus placebo, with no restrictions on treatment duration (Katsiki et al., 2013); Outcome measures: reduction in SUA levels and incidence of gout (Kodama et al., 2009); Exclusion criteria: 1) reviews; 2) literature not available in full text; 3) studies with insufficient data for extraction; 4) animal studies; 5) duplicate publications; and 6) nonrandomized controlled trials (nRCTs).

### 2.3 Data extraction

In accordance with the established inclusion and exclusion criteria, two reviewers (S.Y. and Q.H.) independently evaluated the retrieved literature. Disagreement was resolved through discussion with a third reviewer (N.S.), and consensus was reached for final decisions. The data extraction included methodological quality; publication details (title, author, publication date, country, and clinical trial registration code); patient characteristics (sex, age, number of cases in each group, intervention measures, and duration of treatment); outcome indicators of interest; and relevant outcome measurement data (Serum urate level at baseline and gout incidence).

### 2.4 Quality assessment

The risk of bias for each included RCT was assessed using the Cochrane Risk of Bias tool version 2 (ROB 2) (Higgins et al., 2024). The tool evaluates bias arising from the randomization process, deviations from intended interventions, missing outcome data, measurement of the outcome, selection of the reported result, and overall risk of bias. Each criterion was rated as “High”, “Low”, or “Some concerns” based on study specifics. The Grading of Recommendations Assessment, Development and Evaluation (GRADE) tool was used to assess outcome evidence quality.

### 2.5 Statistical analyses

Statistical analysis was performed using RevMan 5.4 software. The relative risk (RR) and 95% confidence interval (CI) were calculated for dichotomous (gout incidence). Mean difference (MD) and 95% CI were reported for continuous outcomes. Heterogeneity was assessed using the χ^2^ test and *I* ^2^ statistic. A fixed-effects model was used if *I* ^2^ was <50%. Otherwise, a random-effects model was utilized when *I* ^2^ was ≥50%. Due to variability in the types of control across studies, a random-effects model was adopted for conservative analysis. Statistical significance was set at *P* < 0.05. Subgroup analyses were performed based on 1) type of SGLT2 inhibitors and 2) patient populations (e.g., T2DM, non-DM, T1DM). Publication bias was assessed using funnel plots. Sensitivity analyses were conducted to ensure the stability of the conclusions.

## 3 Results

### 3.1 Study search and trial characteristics

**FIGURE 1:**
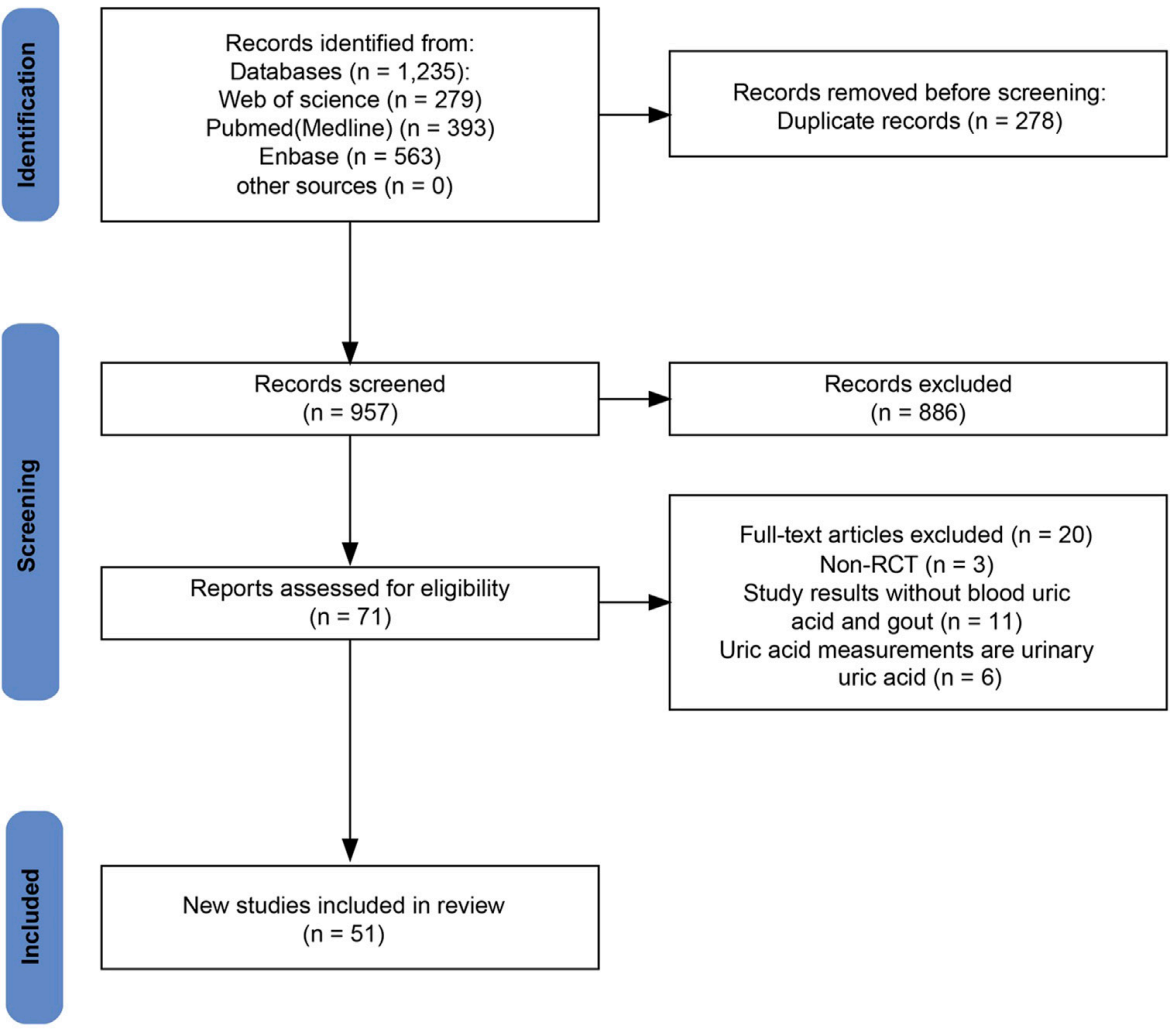
Flow diagram for study identification and inclusion.

The initial search retrieved 1,235 studies, of which 957 were unique after duplicate removal. After screening titles and abstracts, 886 studies were excluded. Full-text assessment led to the exclusion of an additional 20 studies due to issues related to outcomes, study types, and outcome measures. Ultimately, 51 RCTs involving a total of 54,544 patients were included in the meta-analysis (Anker et al., 2021; Kondo et al., 2023; Lee MMY. et al., 2021; Ramírez-Rodríguez et al., 2020; Refardt et al., 2020; Verma et al., 2022; Zanchi et al., 2022). The specific literature search process is shown in Figure 1.

Baseline characteristics of the 51 included studies are summarized in Table 1. The included studies assessed eight different SGLT2 inhibitors: canagliflozin, dapagliflozin, empagliflozin, ipragliflozin, luseogliflozin, sotagliflozin, tofogliflozin, and bexagliflozin. All included studies were placebo-controlled RCTs. The distribution of patient enrollment across the studies was as follows: 6 studies (n = 12,381) evaluated canagliflozin, 16 studies (n = 15,276) evaluated dapagliflozin, 16 studies (n = 21,904) evaluated empagliflozin, four studies (n = 573) evaluated ipragliflozin, four studies (n = 734) evaluated luseogliflozin, two studies (n = 2977) evaluated sotagliflozin, two studies (n = 382) evaluated tofogliflozin, and 1 study (n = 317) evaluated bexagliflozin, totaling 54,544 patients. The mean age of participants ranged from 31.80 to 76.00 years, and the proportion of males varied between 25.00% and 82.60%. The follow-up duration ranged from 4 days to 338 weeks. Seven studies included patients without T2DM (Anker et al., 2021; Kondo et al., 2023; Lee MMY. et al., 2021; Ramírez-Rodríguez et al., 2020; Refardt et al., 2020; Verma et al., 2022; Zanchi et al., 2022), and the remainder included T2DM patients. Any company or program did not sponsor eleven of the studies included in the literature, whereas all the remaining studies received sponsorship.

**TABLE 1:** Baseline characteristics of the included studies (n = 51).

Author	Register number/Trial name	Study type	No. of patients(n)	N	Intervention	Mean age (years) (mean ± SD)	Males (%)	Mean BMI (kg/m^2^)	Duration of diabetes (years) (mean ± SD)	Mean serum urate level at baseline (μmol/L) (mean ± SD)	Length of follow-up	Funding
Li et al. (2020)	NCT01032629/NCT01989754	RCT	10142	5795	Cana 100 mg and 300 mg	63.20 ± 8.30	65.00%	31.90 ± 5.90	13.50 ± 7.70	348.20 ± 94.30	338 weeks	Janssen Research and Development
				4347	PLA	63.40 ± 8.20	63.00%	32.00 ± 6.00	13.70 ± 7.80	349.80 ± 97.10		
Ferreira et al. (2022)	NCT01131676	RCT	7028	2347	Empa 10 mg	63.00 ± 8.60	70.50%	30.60 ± 5.20	30.60 ± 5.20	351.65 ± 1.78	206 weeks	Boehringer Ingelheim
				2344	Empa 25 mg	63.20 ± 8.60	71.90%	30.60 ± 5.30	30.60 ± 5.30	354.62 ± 1.78		
				2337	PLA	63.20 ± 8.80	72.00%	30.70 ± 5.20	30.70 ± 5.20	357.60 ± 1.78		
Verma et al. (2022)	NCT03057977	RCT	3730	1863	Empa 10 mg	67.20 ± 10.80	76.50%	NR	NR	NR	100 weeks	Boehringer Ingelheim
				1867	PLA	66.50 ± 11.20	75.60%	NR	NR	NR		
Anker et al. (2021)	NCT03057951	RCT	5988	2997	Empa 10 mg	71.80 ± 9.30	55.40%	NR	NR	NR	52 weeks	Boehringer Ingelheim
				2991	PLA	71.90 ± 9.60	55.30%	NR	NR	NR		
Kondo et al. (2023)	NCT03036124	RCT	4744	2373	Dapa 10 mg	66.20 ± 11.00	76.20%	NR	NR	NR	94 weeks	AstraZeneca
				2371	PLA	66.50 ± 10.80	77.00%	NR	NR	NR		
	NCT03619213	RCT	6263	3131	Dapa 10 mg	71.80 ± 9.60	56.40%	NR	NR	NR	124 weeks	AstraZeneca
				3132	PLA	71.50 ± 9.50	55.80%	NR	NR	NR		
Strojek et al. (2011)	NCT00680745	RCT	438	142	Dapa 5 mg	60.20 ± 9.73	50.00%	29.84 ± 5.20	7.40 ± 5.70	303.90 ± 79.80	24 weeks	AstraZeneca
				151	Dapa 10 mg	58.90 ± 8.32	43.70%	29.75 ± 5.60	7.20 ± 5.50	301.00 ± 82.40		
				145	PLA	60.30 ± 10.20	49.00%	29.74 ± 4.60	7.40 ± 5.70	315.20 ± 93.60		
Rosenstock et al. (2012a)	NCT00642278	RCT	193	64	Cana 100 mg	51.70 ± 8.00	56.00%	NR	6.10 ± 4.70	NR	12 weeks	Janssen Research and Development, LLC
				64	Cana 300 mg	52.30 ± 6.90	56.00%	NR	5.90 ± 5.20	NR		
				65	PLA	53.30 ± 7.80	48.00%	NR	6.40 ± 5.00	NR		
Rosenstock et al. (2012b)	NCT00683878	RCT	420	141	Dapa 5 mg	53.20 ± 10.90	55.30%	NR	5.64 ± 5.36	NR	24 weeks	Bristol-Myers Squibb and AstraZeneca
				140	Dapa 10 mg	53.80 ± 10.40	42.10%	NR	5.75 ± 6.44	NR		
				139	PLA	53.50 ± 11.40	51.10%	NR	5.07 ± 5.05	NR		
Bailey et al. (2013)	NCT00528879	RCT	409	137	Dapa 5 mg	54.30	50.40%	NR	NR	323.00 ± 88.00	102 weeks	AstraZeneca
				135	Dapa 10 mg	52.70	57.00%	NR	NR	323.00 ± 80.00		
				137	PLA	53.70	55.50%	NR	NR	314.00 ± 79.00		
Bode et al. (2013)	NCT01106651	RCT	714	241	Cana 100 mg	64.30 ± 6.50	51.50%	31.40 ± 4.40	12.30 ± 7.80	339.10	26 weeks	Janssen Research and Development, LLC
				236	Cana 300 mg	63.40 ± 6.00	54.70%	31.50 ± 4.60	11.30 ± 7.20	341.40		
				237	PLA	63.20 ± 6.20	60.30%	31.80 ± 4.80	11.40 ± 7.30	343.40		
Häring et al. (2013)	NCT01159600	RCT	666	225	Empa 10 mg	57.00 ± 9.20	50.00%	28.30 ± 5.40	NR	314.00 ± 127.00	24 weeks	Boehringer Ingelheim
				216	Empa 25 mg	57.40 ± 9.30	53.00%	28.30 ± 5.50	NR	298.00 ± 115.00		
				225	PLA	56.90 ± 9.20	50.00%	27.90 ± 4.90	NR	307.00 ± 110.00		
Roden et al. (2013)	NCT01177813	RCT	676	224	Empa 10 mg	56.20 ± 11.60	63.00%	28.30 ± 5.50	NR	293.00 ± 109.00	24 weeks	Boehringer Ingelheim and Eli Lilly
				224	Empa 25 mg	53.80 ± 11.60	65.00%	28.20 ± 5.50	NR	297.00 ± 124.00		
				228	PLA	54.90 ± 10.90	54.00%	28.70 ± 6.20	NR	307.00 ± 133.00		
Stenlöf et al. (2013)	NCT01081834	RCT	584	195	Cana 100 mg	55.10 ± 10.80	41.50%	31.30 ± 6.60	4.50 ± 4.40	320.00	26 weeks	Janssen Research and Development, LLC
				197	Cana 300 mg	55.30 ± 10.20	45.20%	31.70 ± 6.00	4.30 ± 4.70	326.30		
				192	PLA	55.70 ± 10.90	45.80%	31.80 ± 6.20	4.20 ± 4.10	333.10		
Wilding et al. (2013a)	NCT01106625	RCT	469	157	Cana 100 mg	57.40 ± 10.50	48.40%	33.30 ± 6.30	9.00 ± 5.70	322.30	52 weeks	Janssen Research and Development, LLC
				156	Cana 300 mg	56.10 ± 8.90	55.80%	33.20 ± 6.30	9.40 ± 6.40	340.10		
				156	PLA	56.80 ± 8.30	48.70%	32.70 ± 6.80	10.30 ± 6.70	332.90		
Wilding et al. (2013b)	NCT01117584	RCT	201	68	Ipra 50 mg	58.60 ± 7.60	47.10%	31.10 ± 4.90	6.00 ± 5.30	NR	12 weeks	Janssen Research and Development, LLC
				67	Ipra 100 mg	58.10 ± 8.20	56.70%	31.80 ± 5.20	5.70 ± 4.80	NR		
				66	PLA	57.30 ± 8.60	54.50%	32.00 ± 4.80	5.70 ± 3.20	NR		
Kadowaki et al. (2014)	NCT01193218	RCT	437	109	Empa 10 mg	57.90 ± 9.40	70.60%	25.30 ± 4.40	NR	277.00 ± 124.00	12 weeks	Boehringer Ingelheim
				109	Empa 25 mg	57.20 ± 9.70	77.10%	25.10 ± 3.80	NR	277.00 ± 101.00		
				110	Empa 50 mg	56.60 ± 10.30	77.30%	25.00 ± 3.60	NR	262.00 ± 136.00		
				109	PLA	58.70 ± 8.70	73.40%	25.60 ± 3.40	NR	271.00 ± 127.00		
Kashiwagi et al. (2014)	NCT00621868	RCT	213	72	Ipra 50 mg	55.90 ± 11.40	59.72%	25.80 ± 3.50	6.60 ± 6.80	NR	12 weeks	Astellas Pharma
				72	Ipra 100 mg	56.00 ± 10.40	68.00%	25.90 ± 3.80	7.80 ± 7.30	NR		
				69	PLA	55.20 ± 9.70	71.00%	25.10 ± 3.40	6.30 ± 5.50	NR		
Qiu et al. (2014)	NCT01340664	RCT	279	93	Cana 100 mg	58.60 ± 8.90	43.00%	33.00 ± 7.00	6.70 ± 4.90	310.70	18 weeks	Janssen Research and Development, LLC
				93	Cana 300 mg	56.70 ± 10.30	47.30%	32.30 ± 6.80	7.30 ± 6.00	323.80		
				93	PLA	57.00 ± 9.30	49.50%	32.30 ± 5.70	7.00 ± 6.40	322.80		
Barnett et al. (2014)	NCT01164501	RCT	738	98	Empa 10 mg (1)	63.20 ± 8.50	61.20%	NR	NR	341.00 ± 126.00	52 weeks	Boehringer Ingelheim, Eli Lilly
				97	Empa 25 mg (1)	62.00 ± 8.40	62.90%	NR	NR	337.00 ± 159.00		
				95	PLA (1)	62.60 ± 8.10	58.90%	NR	NR	339.00 ± 125.00		
				187	Empa 25 mg (2)	64.60 ± 8.90	57.20%	NR	NR	419.00 ± 158.00		
				187	PLA (2)	65.10 ± 8.20	56.70%	NR	NR	439.00 ± 153.00		
				37	Empa 25 mg (3)	65.40 ± 10.20	56.80%	NR	NR	559.00 ± 126.00		
				37	PLA (3)	62.90 ± 11.90	51.40%	NR	NR	583.00 ± 162.00		
Bolinder et al. (2012)	NCT00855166	RCT	180	91	Dapa 10 mg	60.60 ± 8.20	55.10%	32.10 ± 3.90	6.00 ± 4.50	346.80 ± 68.90	24 weeks	No Funding
				89	PLA	60.80 ± 6.90	56.00%	31.70 ± 3.90	5.50 ± 5.30	338.40 ± 61.70		
Eriksson et al. (2018)	NCT02279407	RCT	84	21	Dapa 10 mg (1)	65.00 ± 6.50	76.19%	30.50 ± 2.80	6.70 ± 6.00	373.00 ± 69.00	12 weeks	AstraZeneca
				21	PLA (1)	65.00 ± 5.50	80.95%	30.30 ± 3.10	6.50 ± 4.20	365.00 ± 75.00		
				22	Dapa 10 mg (2)	65.00 ± 5.40	68.18%	30.50 ± 2.80	8.50 ± 4.50	344.00 ± 78.00		
				20	PLA (2)	65.00 ± 5.60	55.00%	33.0 ± 4.10	6.30 ± 5.10	370.00 ± 83.00		
Ji et al. (2014)	NCT01095653	RCT	393	128	Dapa 5 mg	53.00 ± 11.10	65.60%	25.17 ± 3.29	1.15 ± 2.30	309.40 ± 71.4	24 weeks	No Funding
				133	Dapa 10 mg	51.20 ± 9.89	64.70%	25.76 ± 3.43	1.67 ± 2.80	297.50 ± 77.35		
				132	PLA	49.90 ± 10.87	65.90%	25.93 ± 3.64	1.30 ± 2.00	321.30 ± 95.20		
Kaku et al. (2014)	Japic CTI-101349	RCT	171	57	Tofo 10 mg	58.60 ± 9.80	66.70%	25.07 ± 3.53	6.30 ± 7.10	283.82 ± 60.10	24 weeks	No Funding
				58	Tofo 20 mg	56.60 ± 10.20	67.20%	24.99 ± 4.55	6.40 ± 5.10	298.95 ± 70.80		
				56	PLA	56.80 ± 9.90	66.10%	26.00 ± 4.11	6.00 ± 6.10	302.85 ± 82.70		
Kario et al. (2019)	NCT03050229	RCT	131	68	Empa 10 mg	70.90 ± 8.70	52.90%	26.10 ± 3.80	NR	321.30 ± 89.25	12 weeks	Boehringer Ingelheim and Eli Lilly and Company Diabetes Alliance
				63	PLA	69.30 ± 7.80	52.40%	26.00 ± 4.90	NR	321.30 ± 89.25		
Kashiwagi et al. (2015)	NCT01057628	RCT	129	62	Ipra 50 mg	60.60 ± 9.40	67.70%	25.30 ± 3.10	7.53 ± 6.88	289.17 ± 65,45	16 weeks	No Funding
				67	PLA	58.30 ± 10.50	71.60%	25.60 ± 3.90	5.90 ± 5.09	272.51 ± 73.18		
Kohan et al. (2014)	NCT00663260	RCT	252	83	Dapa 5 mg	66.00 ± 8.90	66.30%	59.00 ± 71.10	16.90 ± 9.00	434.35 ± 126.14	104 weeks	No Funding
				85	Dapa 10 mg	68.00 ± 7.70	65.90%	54.00 ± 63.50	18.20 ± 10.10	424.23 ± 101.74		
				84	PLA	67.00 ± 8.60	63.10%	50.00 ± 59.50	15.70 ± 9.50	419.47 ± 15.43		
Kovacs et al. (2015)	NCT01210001	RCT	498	165	Empa 10 mg	54.70 ± 9.90	50.30%	29.20 ± 5.60	NR	288.00 ± 116.00	24 weeks	Boehringer Ingelheim and Eli Lilly and Company
				168	Empa 25 mg	54.20 ± 8.90	50.60%	29.10 ± 5.50	NR	271.00 ± 117.00		
				165	PLA	54.60 ± 10.50	44.20%	29.30 ± 5.40	NR	275.00 ± 113.00		
Lee et al. (2021a)	NCT03485092	RCT	105	52	Empa 10 mg	68.20 ± 11.70	65.40%	30.90 ± 5.90	NR	391.60 ± 132.50	36 weeks	Boehringer Ingelheim
				53	PLA	69.20 ± 10.60	81.10%	30.40 ± 5.10	NR	405.80 ± 106.30		
Lee et al. (2021b)	NCT02459353	RCT	84	41	Dapa 10 mg	59.70 ± 8.00	41.50%	27.30 ± 3.90	15.10 ± 7.20	273.10 ± 77.35	12 weeks	No Funding
				43	PLA	57.70 ± 7.30	41.90%	26.60 ± 3.00	15.10 ± 6.00	268.94 ± 74.37		
Mozawa et al. (2021)	UMIN000030158	RCT	96	46	Empa 10 mg	63.90 ± 10.40	82.60%	25.20 ± 3.70	3.19 ± 3.62	345.10 ± 83.30	24 weeks	Boehringer Ingelheim and Eli Lilly and Company
				50	PLA	64.60 ± 11.60	78.00%	25.20 ± 4.10	2.70 ± 3.61	339.15 ± 89.25		
Pollock et al. (2019)	NCT02547935	RCT	293	145	Dapa 10 mg	64.70 ± 8.60	70.00%	30.19 ± 5.30	17.55 ± 7.70	399.40 ± 98.90	24 weeks	AstraZeneca
				148	PLA	64.70 ± 8.50	71.00%	30.34 ± 5.60	17.71 ± 9.50	414.90 ± 92.60		
Ramírez-Rodríguez et al. (2020)	NCT02700334	RCT	24	12	Dapa 10 mg	51.50 ± 6.30	33.33%	30.30 ± 3.50	NR	334.00 ± 70.00	12 weeks	No Funding
				12	PLA	46.70 ± 9.80	25.00%	33.00 ± 2.20	NR	312.00 ± 101.00		
Refardt et al. (2020)	NCT02874807	RCT	87	43	Empa 25 mg	74.00 ± 14.00	37.00%	24.00 ± 4.10	NR	214.00 ± 37.00	4 days	Schweizerischer Nationalfonds zur Förderung der Wissenschaftlichen Forschung
				44	PLA	76.00 ± 12.00	36.00%	23.10 ± 4.90	NR	181.00 ± 30.25		
Ross et al. (2015)		RCT	535	214	Empa 25 mg	58.20 ± 10.20	53.30%	32.10 ± 5.30	NR	328.00 ± 122.00	16 weeks	Boehringer Ingelheim and Eli Lilly and Company
				214	Empa 10 mg	58.50 ± 10.80	50.50%	31.90 ± 5.40	NR	327.00 ± 131.00		
				107	PLA	57.90 ± 11.20	51.40%	32.00 ± 5.00	NR	330.00 ± 115.00		
Schumm-Draeger et al. (2015)	NCT01217892	RCT	299	99	Dapa 5 mg	55.30 ± 9.30	46.50%	33.09 ± 4.94	5.12 ± 4.20	331.93 ± 81.96	16 weeks	Bristol-Myers Squibb and AstraZeneca
				99	Dapa 10 mg	58.50 ± 9.80	49.50%	32.25 ± 5.01	5.45 ± 4.05	349.77 ± 89.81		
				101	PLA	58.50 ± 9.40	46.50%	31.74 ± 4.69	5.53 ± 4.23	337.28 ± 87.50		
Seino et al. (2014a)	JapicCTI-090908	RCT	176	61	Luse 2.5 mg	58.30 ± 9.40	57.40%	24.80 ± 3.56	6.15 ± 6.5	302.85 ± 88.65	12 weeks	Taisho Pharmaceutical Co. Ltd.
				61	Luse 5 mg	56.80 ± 9.30	72.10%	24.50 ± 3.21	5.77 ± 5.55	302.26 ± 76.75		
				54	PLA	76.00 ± 11.00	74.10%	25.20 ± 4.26	7.30 ± 6.43	317.73 ± 77.25		
Seino et al. (2014b)	Japic CTI-101191	RCT	167	56	Luse 2.5 mg	57.40 ± 9.30	67.90%	24.79 ± 3.81	4.60 ± 4.40	307.02 ± 76.16	12 weeks	Taisho Pharmaceutical Co. Ltd.
				54	Luse 5 mg	57.30 ± 11.40	75.90%	26.43 ± 4.26	4.50 ± 4.20	296.90 ± 67.23		
				57	PLA	57.10 ± 10.0	71.90%	25.15 ± 3.62	5.10 ± 4.60	311.78 ± 60.09		
Seino et al. (2014c)	JapicCTI-111661	RCT	158	79	Luse 2.5 mg	58.90 ± 10.10	75.90%	25.98 ± 4.88	6.50 ± 5.90	308.21 ± 70.21	6 weeks	Taisho Pharmaceutical Co. Ltd.
				79	PLA	59.60 ± 9.30	70.90%	25.34 ± 4.19	6.10 ± 5.40	295.12 ± 68.42		
Seino et al. (2018)	JapicCTI-142582	RCT	233	159	Luse 2.5 mg	57.40 ± 10.30	70.40%	25.42 ± 3.53	11.70 ± 7.60	280.24 ± 66.64	52 weeks	Taisho Pharmaceutical Co. Ltd.
				74	PLA	57.10 ± 10.90	68.90%	25.15 ± 3.44	12.10 ± 6.80	289.76 ± 70.80		
Søfteland et al. (2017)	NCT01734785	RCT	327	109	Empa 10 mg	54.30 ± 9.60	60.60%	31.20 ± 5.90	NR	301.00 ± 124.00	24 weeks	No Funding
				110	Empa 25 mg	55.40 ± 9.90	64.50%	29.90 ± 5.30	NR	297.00 ± 116.00		
				108	PLA	55.90 ± 9.70	55.60%	29.60 ± 5.70	NR	310.00 ± 118.00		
Terauchi et al. (2017)	NCT02201004	RCT	211	141	Tofo 20 mg	59.10 ± 10.80	63.80%	25.80 ± 3.50	15.02 ± 9.36	300.47 ± 74.37	16 weeks	Sanofi K.K. and Kowa Company, Ltd.
				70	PLA	56.40 ± 10.00	68.60%	26.90 ± 3.90	12.39 ± 7.34	311.18 ± 84.49		
Tikkanen et al. (2015)	NCT01370005	RCT	823	276	Empa 10 mg	60.60 ± 8.50	62.00%	32.40 ± 5.30	NR	341.85 ± 81.78	12 weeks	No Funding
				276	Empa 25 mg	59.90 ± 9.70	56.50%	33.00 ± 5.00	NR	338.27 ± 79.52		
				271	PLA	60.30 ± 8.80	62.00%	32.40 ± 4.90	NR	347.37 ± 82.73		
van Raalte et al. (2019)	NCT02384941/NCT02421510	RCT	1575	524	Sota 200 mg	44.40 ± 13.70	50.60%	28.90 ± 5.60	21.60 ± 12.50	269.53 ± 2.97	52 weeks	Dutch Diabetes Foundation
				525	Sota 400 mg	44.00 ± 13.40	48.20%	28.70 ± 5.20	21.50 ± 12.30	264.77 ± 2.97		
				526	PLA	42.50 ± 13.30	51.50%	28.50 ± 5.30	21.20 ± 12.00	268.34 ± 2.97		
Weber et al. (2016b)	NCT01195662	RCT	449	225	Dapa 10 mg	56.00 ± 6.00	52.00%	NR	7.70 ± 5.90	334.95 ± 92.59	12 weeks	Bristol-Myers Squibb, AstraZeneca
				224	PLA	57.00 ± 6.00	58.00%	NR	7.30 ± 5.00	325.28 ± 78.·92		
Weber et al. (2016a)	NCT01137474	RCT	613	302	Dapa 10 mg	55.60 ± 8.40	59.30%	NR	8.20 ± 6.40	321.3 ± 83.30	12 weeks	BristolMyers Squibb, AstraZeneca, Novartis and Forest Pharmaceuticals
				311	PLA	56.20 ± 8.90	55.00%	NR	7.60 ± 6.20	321.3 ± 77.35		
Yang et al. (2018)	NCT02096705	RCT	272	139	Dapa 10 mg	56.50 ± 8.40	47.50%	26.40 ± 3.80	12.70 ± 7.20	321.30 ± 95.20	24 weeks	No Funding
				133	PLA	58.60 ± 8.90	48.10%	26.70 ± 3.30	12.20 ± 6.70	321.30 ± 89.25		
Zanchi et al. (2022)	NCT03093103	RCT	39	26	Empa 10 mg	31.80 ± 7.40	69.23%	28.50 ± 4.90	NR	303.00 ± 69.40	1 month	Boehringer Ingelheim
				13	PLA	35.30 ± 10.80	61.54%	28.60 ± 4.70	NR	274.00 ± 73.20		
Hao et al. (2018)	ChiCTR1800015830	RCT	59	29	Dapa 10 mg	57.77 ± 12.29	66.67%	27.34 ± 3.88	12.20 ± 6.59	348.33 ± 102.15	3 days after achieving good glycemic control during hospitalization	No Funding
				30	PLA	58.97 ± 10.50	58.62%	25.90 ± 3.51	9.47 ± 5.88	326.21 ± 103.39		
Tanaka et al. (2020)	UMIN000016563	RCT	30	15	Ipra 50 mg	59.10 ± 11.20	53.30%	30.50 ± 7.00	NR	339.8 ± 85.20	12 weeks	Astellas Pharm
				15	PLA	62.50 ± 13.50	46.70%	31.40 ± 5.10	NR	331.9 ± 73.30		
Halvorsen et al. (2023)	NCT03259789	RCT	317	158	Bexa 20 mg	56.00 ± 10.10	63.30%	29.70 ± 6.50	9.31 ± 6.60	311.00 ± 79.00	24 weeks	Theracos Sub, LLC
				159	PLA	55.60 ± 11.20	59.10%	30.00 ± 6.30	8.88 ± 5.90	296.00 ± 77.00		
Sridhar et al. (2023)	NCT02531035	RCT	1402	699	Sota 400 mg	NR	NR	NR	NR	265.50 ± 73.30	24 weeks	Lexicon Pharmaceuticals, Inc. V.S.S
				703	PLA	NR	NR	NR	NR	264.00 ± 76.00		

Footnotes: RCT: randomized controlled trial; NR: no report; BMI: body mass index; PLA: placebo; Cana: canagliflozin; Empa: empagliflozin; Dapa: dapagliflozin; Ipra: ipragliflozin; Tofo: tofacogliflozin; Luse: luseogliflozin; Sota: sotagliflozin.

### 3.2 Assessment of the quality of the included studies

All included studies were described as randomized. Detailed risk of bias assessments are presented in Supplementary Table 1. Overall, the risk of bias across the included literature was judged to be predominantly low. For instance, regarding the randomization domain, 43 studies were judged as ‘low risk’ and eight studies as ‘some concerns’. None were categorized as high risk. Missing outcome data was the main factor contributing to potential bias in these RCTs.

### 3.3 Meta-analyses of SUA changes

**FIGURE 2:**
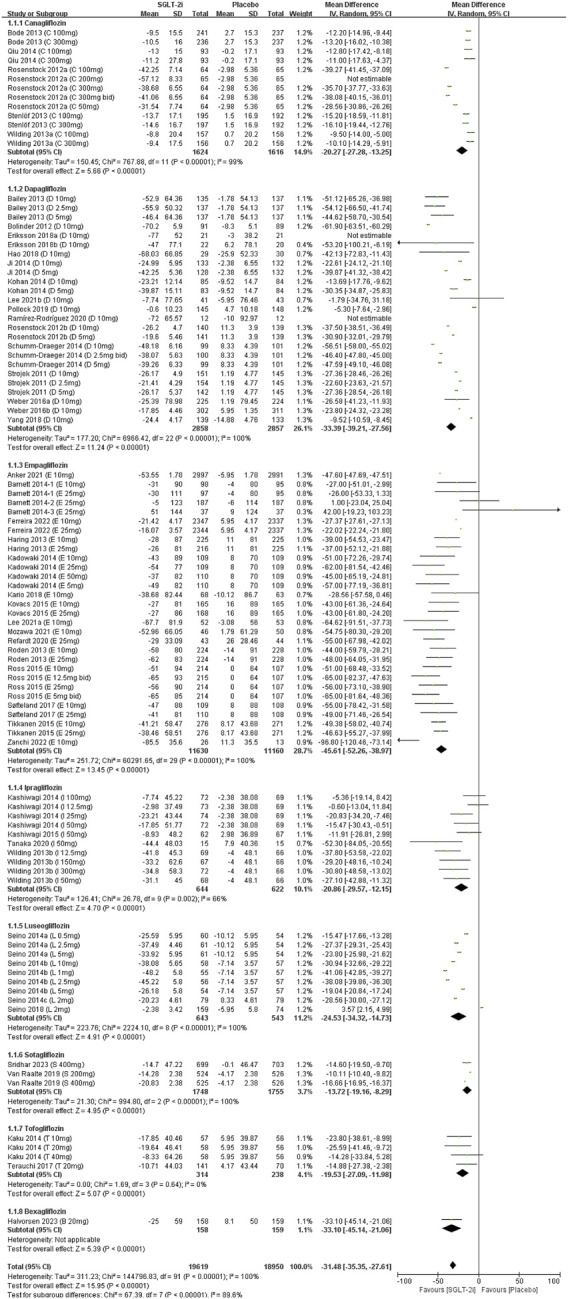
Forest plots for the comparison of SGLT-2i vs. Placebo on change of SUA by MD analysis.

Data on SUA change from all fifty-one studies were pooled for meta-analysis. Overall, SGLT2 inhibitors significantly reduced SUA levels compared to placebo [MD = −32.14 μmol/L, 95% CI (−35.96 to −28.31); *P* < 0.001]. However, heterogeneity was high (*I* ^2^ = 100%, *P* < 0.00001). Thus, a random-effect model was applied (Figure 2). The evidence quality was rated as low, downgraded to one level for inconsistency, one level for indirectness, and one level for imprecision (Table 2).

**TABLE 2:** The GRADE profiles: SGLT2 inhibitors compared to placebo in the change of gout.

Outcomes	Illustrative comparative risks (95% CI)		No of participants (studies)	Quality assessment						Quality of the evidence (GRADE)
	Assumed risk	Corresponding risk		Design	Risk of bias	Inconsistency	Indirectness	Imprecision	Other considerations	
	Placebo	SGLT2								
The change of gout		The mean the change of gout in the intervention groups was 32.14 lower (35.96–28.31 lower)	19717 (51 studies)	RCT	No serious risk of bias	Serious^a^	No serious indirectness	Serious^b^	None	⊕⊕⊝⊝ low^a^ ^,^ ^b^

CI: Confidence interval.

Low quality: Further research is very likely to have an important impact on our confidence in the estimate of effect and is likely to change the estimate.

^a^ Downgraded one level for inconsistency (Substantial heterogeneity was present among the studies (I2 = 100%).

^b^ Downgraded one level for imprecision (Very small samples sizes in Ramírez-Rodríguez et al., 2020).

In subgroup analyses of SGLT2 inhibitors, participants receiving all types of SGLT2 inhibitors demonstrated statistically significant reductions in SUA compared to the placebo group. Empagliflozin had the most effect on SUA reduction [MD = −45.61 μmol/L, 95% CI (−52.26 to −38.97); *P* < 0.00001], whereas sotagliflozin had the least effect on SUA levels [MD = −13.72 μmol/L, 95% CI (−19.16 to −8.29); *P* < 0.00001] (Supplementary Table 2).

Subgroup analyses by patient population, revealed significant SUA reduction across all groups of SGLT2 inhibitors compared to placebo. The patients without DM had the most effect on SUA reduction [MD = −92.66 μmol/L, 95% CI (−114.86 to −70.45); *P* < 0.00001], and smallest in patients with T1DM [MD = −14.60 μmol/L, 95% CI (−19.50 to −9.70); *P* < 0.00001]. Patients with T2DM showed an intermediate effect on SUA levels (Supplementary Table 2).

### 3.4 Meta-analyses of gout incidence

**FIGURE 3:**
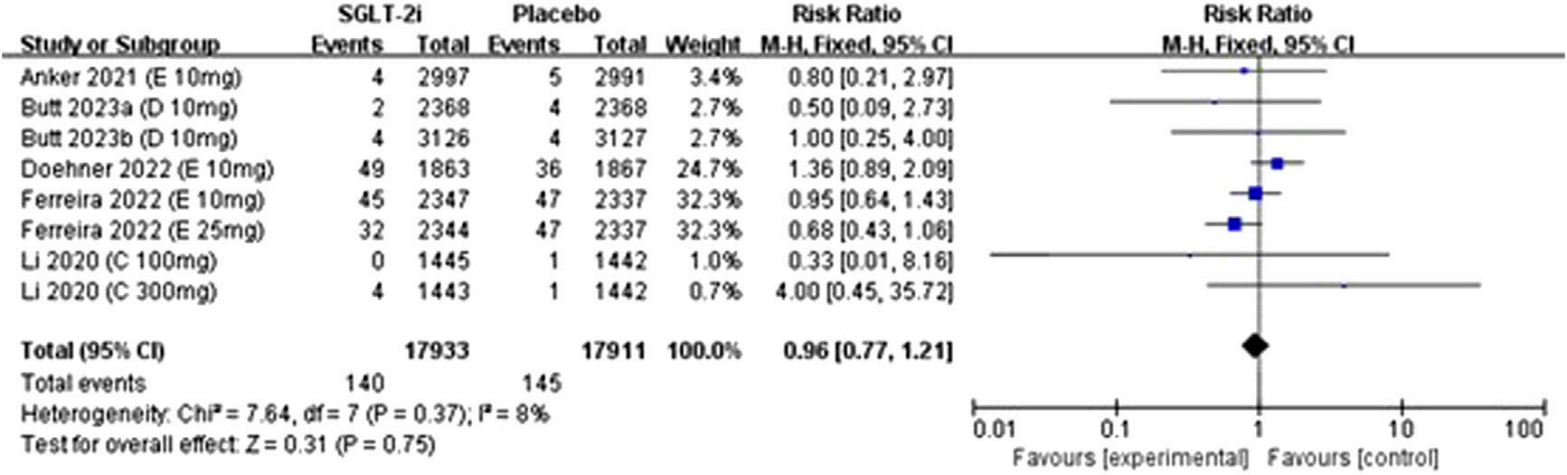
Forest plots for the comparison of SGLT-2i vs. Placebo on incedence of gout by RR analysis.

Eight studies reported data on gout incidence, including trials on empagliflozin (4 studies), dapagliflozin (2 studies), and canagliflozin (2 studies), encompassing a total of 35,844 patients. There was no statistical heterogeneity among the studies (*I* ^*2*^ = 8%, *P* = 0.37), and effect sizes were analyzed via a fixed-effects model with pooled effect sizes. The meta-analysis showed no significant difference in the incidence of gout between SGLT2 inhibitors and placebo groups [RR: 0.96, 95% CI: 0.77 to 1.21; *P* = 0.75]. The details can be seen in Figure 3.

### 3.5 Assessment of publication bias

**FIGURE 4:**
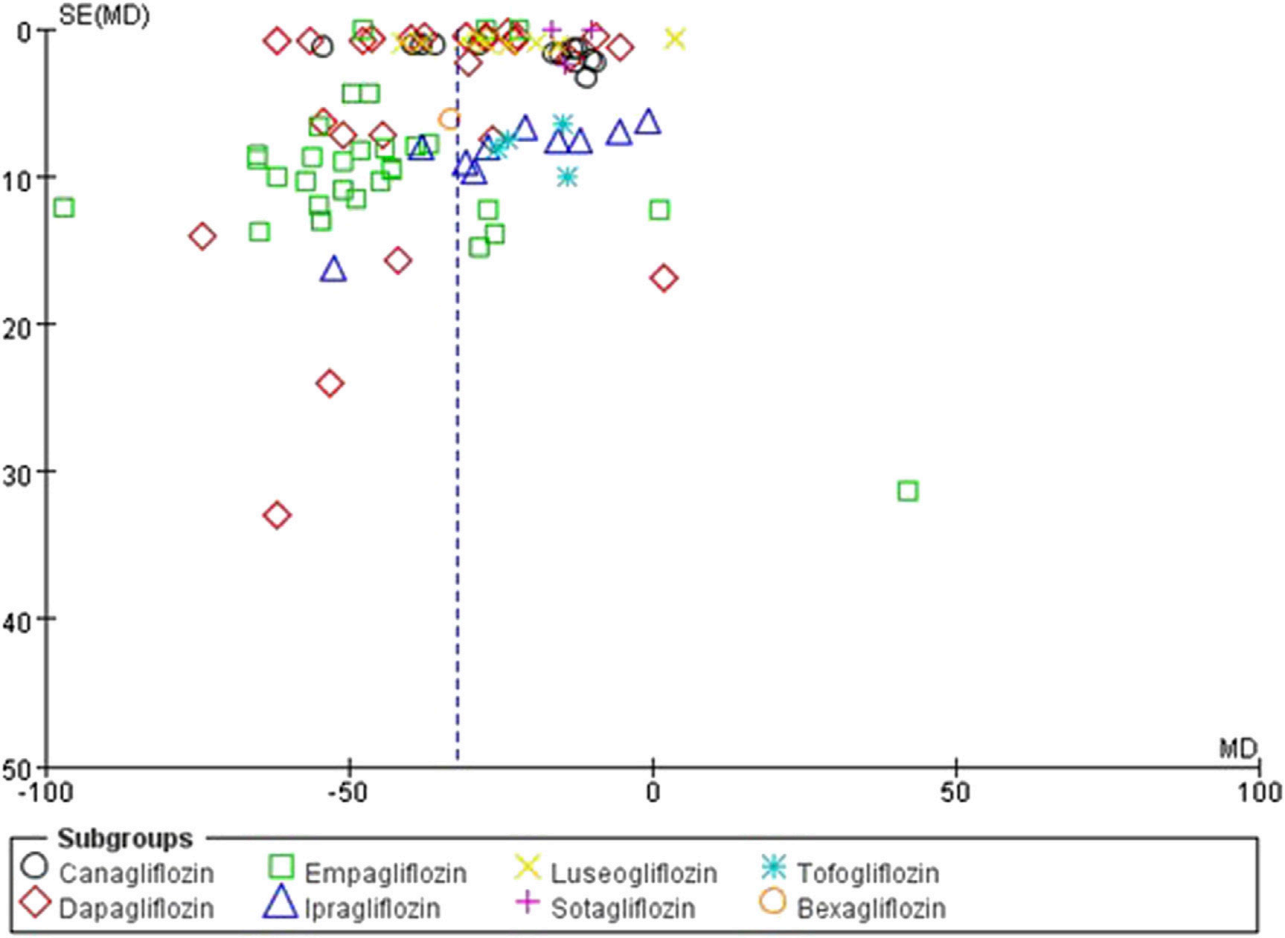
Inverted funnel plot comparing serum uric acid reduction between SGLT2 inhibitors and placebo.

**FIGURE 5:**
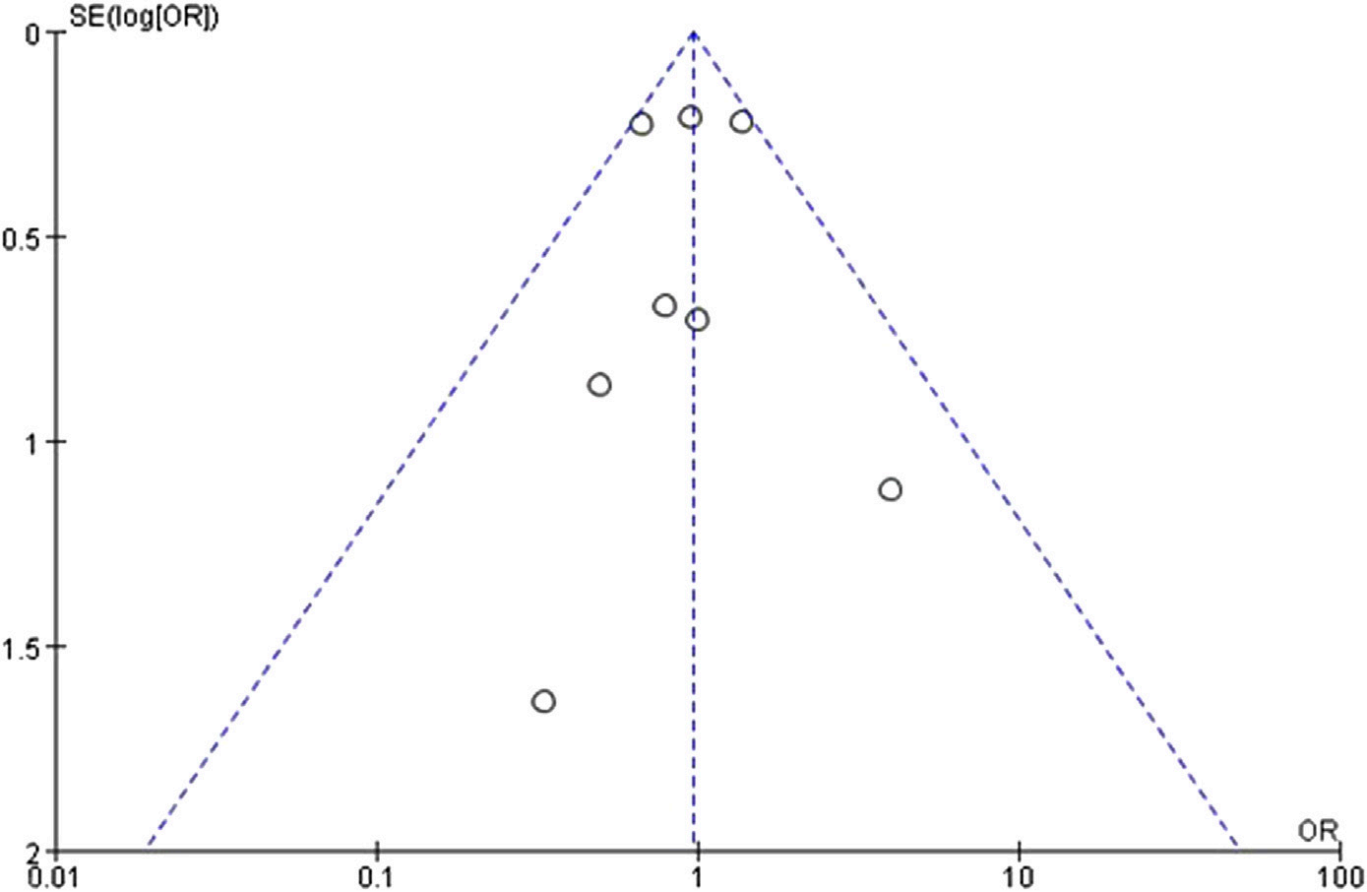
Funnel plot comparing gout incidence between SGLT2 inhibitors and placebo.

The funnel plots were constructed for both the SUA change levels and gout incidence outcome to assess potential publication bias. The observation reveals a symmetrical distribution of the funnel plot, with the majority of studies located at the top, and analysis using Egger’s test resulted in *P* = 0.79, which is greater than 0.05, suggesting a low risk of publication bias (Figure 4). Similarly, the funnel plot of the incidence of gout in patients showed reasonable symmetry, indicating no obvious evidence of publication bias (Figure 5). According to the sensitivity analysis, removing most individual studies did not significantly alter the overall meta-analysis result for SUA reduction; therefore, the study findings are considered robust.

## 4 Discussion

The interventions investigated were SGLT2 inhibitors for the SUA reduction in this review. We found that SGLT2 inhibitors had potential applications in the treatment of hyperuricemia and gout (Otani et al., 2020; Tao et al., 2023). Beyond their clinically recognized glucose-lowering effects, SGLT2 inhibitors have also demonstrated the ability to reduce SUA levels (Wei et al., 2023). This could be especially beneficial for patients with cardiovascular disease or CKD, as elevated SUA levels are commonly observed in these populations (Yuan et al., 2024). SGLT2 inhibitors could offer additional therapeutic benefits for these populations by promoting UA excretion. More importantly, the clinical evidence suggests that the UA-lowering effect of SGLT2 inhibitors remains preserved whether the patient is taking conventional traditional UA-lowering drugs, such as allopurinol, febuxostat, or verinurad (McDowell et al., 2022). Moreover, among adults with asymptomatic hyperuricemia, combining either verinurad or febuxostat with dapagliflozin augmented the SUA-lowering effect more than either agent alone (Stack et al., 2021). Overall, this evidence supports that the addition of SGLT2 inhibitors, in concert with first-line UA-lowering drugs, represents a novel and potentially effective way to manage patients with gout.

While the precise mechanisms of action of SGLT2 inhibitors on SUA are not entirely understood, based customarily on preclinical studies, a number of hypotheses are available. Under normal physiological conditions, UA levels are regulated primarily by renal tubular reabsorption and excretion (Fathallah-Shaykh and Cramer, 2014). SGLT2 inhibitors increase the excretion of urinary glucose and sodium in the urine by reducing their reabsorption in the renal tubules. This leads to a greater urine volume, which helps remove UA and lowers its levels (Kochanowska et al., 2023; Suijk et al., 2022). Another possible explanation is that SGLT2 inhibitors not only promote urinary glucose excretion, but also affect renal tubular urate transporters, such as URAT1 and GLUT9 (Mende, 2015). GLUT9 protein, a glucose transporter, plays a critical role in the reabsorption of both glucose and urate in the renal tubules (Bobulescu and Moe, 2012), with its transport activity for urate being 45 to 60 times higher than for glucose (Shen et al., 2024). It is possible that SGLT2 inhibitors increase urinary glucose excretion, and when glucose concentrations rise in the lumen, the competitive binding of urinary glucose to GLUT9 may inhibit UA reabsorption, resulting in increased UA excretion (Dong et al., 2023). However, canagliflozin maintained SUA reduction in GLUT9-knockout mice (Novikov et al., 2019), indicating alternative pathways including direct transporter effects (Hou et al., 2020) and improvements in overall renal function (Di Costanzo et al., 2023). It is important that these conclusions from animal models cannot usually translate into humans, and there is no clinical validation of these mechanisms directly. Further human studies are needed to clarify the underlying pathways. In addition, recent studies are shedding new light on gout, pointing to a twofold metabolic imbalance at its core: excessive purine biosynthesis via the pentose phosphate pathway coupled with impaired renal/intestinal SUA excretion (Zhang et al., 2022). This process is amplified by coordinated dysregulation of nutritional signaling pathways - upregulation of mTOR/HIF-1α pathways alongside suppression of Sirtuin-1/AMPK activity - which redirects glucose flux toward anabolic metabolism rather than ATP generation (Sant'Ana et al., 2023). This shift in metabolism puts extra strain on the body, driving up oxidative stress that slowly damages heart muscle cells and kidney tubules, eventually paving the way for cardiorenal complications (Aranda-Rivera et al., 2021). Notably, SGLT-2 inhibitors demonstrate dual therapeutic effects by mimicking nutrient-deprived states, reducing pentose phosphate flux (thereby limiting purine/urate synthesis) while enhancing renal UA excretion (Packer, 2024).

This study conducted a meta-analysis of 51 studies to systematically summarize the effects of different classes of SGLT2 inhibitors on SUA-lowering effect and the incidence of gout in patients with or without T2DM. The results revealed that SGLT2 inhibitors significantly reduced SUA compared to placebo, with empagliflozin showing the strongest effect (mean reduction ∼0.9–1.1 mg/dL or 52.3–68.03 μmol/L, approximately 10% from baseline), while sotagliflozin had minimal impact. Concerning gout incidence, although a trend towards reduced risk was observed, no statistically significant difference was found between the SGLT2 inhibitor and placebo groups. However, this analysis was based on few studies (n = 8), low rates of events, varied follow-up durations, which limited the statistical power and precision of the results. Future large-scale adequately powered RCTs which specifically evaluating gout outcomes are needed to confirm the potential role of SGLT2 inhibitors in gout prevention. During data merging, significant heterogeneity was found in the SUA lowering outcome, likely due to racial differences, age variations, different types and doses of SGLT2 inhibitors, and baseline SUA levels. After excluding studies contributing to the significant heterogeneity, reanalysis showed that the comparison results between the SGLT2 inhibitors and placebo groups did not reverse. This indicated that the results were relatively stable.

Although SGLT2 inhibitors have demonstrated significant efficacy in lowering SUA levels, it is important to consider their potential adverse drug reactions. Studies (Matharu et al., 2021; Fitchett, 2019) have shown that SGLT2 inhibitors are associated with adverse drug reactions, such as genital mycotic infections (5% or higher) (Gorgojo-Martínez et al., 2024), urinary tract infections (3%–9%) (Gorgojo-Martínez et al., 2024), diabetic ketoacidosis (0.2%–0.6%) (Bi et al., 2024), and polyuria (2.7%) (Li CX. et al., 2023). Urinary and genital tract infections are the most common adverse drug reactions associated with the use of SGLT2 inhibitors, and most cases are mild to moderate. However, adverse drug reactions such as diabetic ketoacidosis, and hypovolemia are rare but serious and can be fatal if not treated promptly. There is still debate about the relationship between treatment with SGLT2 inhibitors and the incidence of fractures in patients. Considering the SUA-lowering effect and the potential adverse drug reactions, it is important for the clinician to consider the benefits vs. the risks of SGLT2 inhibitors carefully. The treatment choice should be individualized based on the patient’s underlying condition (e.g., diabetes, heart failure) and risk factors. Risks and benefit assessment are important especially in high-risk populations, considering the need to monitor adverse events closely.

Based on the GRADE assessment, the quality of the evidence was downgraded mainly due to three factors: inconsistency, indirectness, and imprecision. Inconsistency arose from the moderate to high heterogeneity observed across the studies, likely due to differences in patient characteristics and treatment durations. Indirectness was a concern because there were few studies that specifically focused on patients without T2DM or those with established gout. Lastly, imprecision was mainly caused by wide confidence intervals in some subgroup analyses and smaller sample sizes in certain comparisons.

While there have been some previous reviews and meta-analyses (Akbari et al., 2022; Sridharan and Alkhidir, 2025; Hu et al., 2022; Li M. et al., 2023) exploring the SUA-lowering effects of SGLT2 inhibitors, our study contributes to the evidence base by utilizing a larger and more current cohort of RCTs, including studies that have been published after 2023 [e.g., Kondo et al. (2023)] which were not included in the prior analyses. Furthermore, we also used more stringent criteria for study inclusion, excluding observational or retrospective studies to reduce bias. A recent 2025 meta-analysis (Sridharan and Alkhidir, 2025) which included 56 RCTs and a total of 16,788 participants, also found empagliflozin to have the most significant SUA-lowering effect, consistent with our findings. However, this meta-analysis included active drugs as controls, and thus, likely introduced a greater degree of heterogeneity, reducing the precision of the estimates for the UA-lowering effects of SGLT2 inhibitors. In contrast, our study focused only on placebo-controlled RCTs and examined a larger sample size (n = 54,544), which provided a less homogeneous cohort and increased the internal validity of our findings. Additionally, unlike prior reviews that were restricted to patients with T2DM, we included patients using SGLT2 inhibitors regardless of their diagnosis. Anyway, we systematically investigated both SUA reduction and gout incidence, contributing to a broader comprehensive evidence base that can be applied in future treatments.

### 4.1 Limitations

The study has several key limitations: 1) Significant heterogeneity among studies may affect result stability; 2) The majority of studies lacked data regarding the incidence of gout, and further assessment is needed to evaluate the impact of SGLT2 inhibitors on gout incidence in patients with and without T2DM; 3) Variations exist in participant age, follow-up duration, and underlying kidney disease types; 4) Insufficient adverse effect data, and long-term safety beyond 52 weeks remains inadequately evaluated; 5) Inclusion limited to placebo-controlled monotherapy RCTs without comparisons between different SGLT2 inhibitors; and 6) The quality of the included studies was limited, raising the possibility of bias; 7) All the included studies were industry-sponsored, which may introduce reporting bias; however, our primary outcome (SUA) is an objective measure and less prone to such bias; 8) Some studies lacked clear ITT analysis, which may introduce bias. These factors warrant cautious interpretation of the findings.

More RCTs are needed to assess the effect of SGLT2 inhibitors in lowering SUA levels and preventing gout, particularly in high-risk patients with established hyperuricemia. Currently, clinical trials, including NCT06674109 (Fernandes, 2024), are evaluating this question. To enhance the quality of future evidence, it is important to declare the amount of UA lowered to achieve the key endpoint in RCTs, and commence definitive monitoring of gout from the beginning of the trial. It is essential that monitor the clinical role of SGLT2 inhibitors in the gout patient population to derive high-quality evidence that could modify future gout management guidelines, similar to the evidence base formed in T2DM, CKD and cardiovascular diseases.

## 5 Conclusion

SGLT2 inhibitors markedly reduce SUA in those with and without T2DM, but the effect on gout incidence is unknown because there is currently limited evidence. More studies are warranted to confirm these results, and assess differences between individual agents.

## Data Availability

The datasets presented in this study can be found in online repositories. The names of the repository/repositories and accession number(s) can be found in the article/Supplementary Material.

## References

[B1] Akbari A. Rafiee M. Sathyapalan T. Sahebkar A. (2022). Impacts of sodium/glucose cotransporter-2 inhibitors on circulating uric acid concentrations: a systematic review and meta-analysis. J. Diabetes Res. 2022, 7520632. 10.1155/2022/7520632 35224108 PMC8872662

[B2] Anker S. D. Butler J. Filippatos G. Ferreira J. P. Bocchi E. Böhm M. (2021). Empagliflozin in heart failure with a preserved ejection fraction. N. Engl. J. Med. 385 (16), 1451–1461. 10.1056/NEJMoa2107038 34449189

[B3] Aranda-Rivera A. K. Cruz-Gregorio A. Aparicio-Trejo O. E. Pedraza-Chaverri J. (2021). Mitochondrial redox signaling and oxidative stress in kidney diseases. Biomolecules 11 (8), 1144. 10.3390/biom11081144 34439810 PMC8391472

[B4] Author Anonymous (2024). Correction to: 2023 Focused Update of the 2021 ESC Guidelines for the diagnosis and treatment of acute and chronic heart failure: developed by the task force for the diagnosis and treatment of acute and chronic heart failure of the European Society of Cardiology (ESC) with the special contribution of the Heart Failure Association (HFA) of the ESC. Eur. Heart J. 45 (1), 53. 10.1093/eurheartj/ehad613 37995350

[B5] Badve S. V. Pascoe E. M. Tiku A. Boudville N. Brown F. G. Cass A. (2020). Effects of allopurinol on the progression of chronic kidney disease. N. Engl. J. Med. 382 (26), 2504–2513. 10.1056/NEJMoa1915833 32579811

[B6] Bailey C. J. Gross J. L. Hennicken D. Iqbal N. Mansfield T. A. List J. F. (2013). Dapagliflozin add-on to metformin in type 2 diabetes inadequately controlled with metformin: a randomized, double-blind, placebo-controlled 102-week trial. BMC Med. 11, 43. 10.1186/1741-7015-11-43 23425012 PMC3606470

[B7] Barnett A. H. Mithal A. Manassie J. Jones R. Rattunde H. Woerle H. J. (2014). Efficacy and safety of empagliflozin added to existing antidiabetes treatment in patients with type 2 diabetes and chronic kidney disease: a randomised, double-blind, placebo-controlled trial. Lancet Diabetes Endocrinol. 2 (5), 369–384. 10.1016/s2213-8587(13)70208-0 24795251

[B8] Bi Y. Chen L. Chen K. Du J. Guo L. Hong T. (2024). Chinese expert consensus on combination therapy of sodium-glucose cotransporter 2 inhibitor and insulin in the treatment of type 2 diabetes mellitus (2023 edition). Chin. J. Diabetes Mellit. 16 (1), 9–19. 10.3760/cma.j.cn115791-20240104-00007

[B9] Bobulescu I. A. Moe O. W. (2012). Renal transport of uric acid: evolving concepts and uncertainties. Adv. Chronic Kidney Dis. 19 (6), 358–371. 10.1053/j.ackd.2012.07.009 23089270 PMC3619397

[B10] Bode B. Stenlöf K. Sullivan D. Fung A. Usiskin K. (2013). Efficacy and safety of canagliflozin treatment in older subjects with type 2 diabetes mellitus: a randomized trial. Hosp. Pract. (1995) 41 (2), 72–84. 10.3810/hp.2013.04.1020 23680739

[B11] Bolinder J. Ljunggren Ö. Kullberg J. Johansson L. Wilding J. Langkilde A. M. (2012). Effects of dapagliflozin on body weight, total fat mass, and regional adipose tissue distribution in patients with type 2 diabetes mellitus with inadequate glycemic control on metformin. J. Clin. Endocrinol. Metab. 97 (3), 1020–1031. 10.1210/jc.2011-2260 22238392

[B12] Chilton R. Tikkanen I. Hehnke U. Woerle H. J. Johansen O. E. (2017). Impact of empagliflozin on blood pressure in dipper and non-dipper patients with type 2 diabetes mellitus and hypertension. Diabetes Obes. Metab. 19 (11), 1620–1624. 10.1111/dom.12962 28387058

[B13] Chino Y. Samukawa Y. Sakai S. Nakai Y. Yamaguchi J. Nakanishi T. (2014). SGLT2 inhibitor lowers serum uric acid through alteration of uric acid transport activity in renal tubule by increased glycosuria. Biopharm. Drug Dispos. 35 (7), 391–404. 10.1002/bdd.1909 25044127 PMC4223977

[B14] Di Costanzo A. Esposito G. Indolfi C. Spaccarotella C. A. M. (2023). SGLT2 inhibitors: a new therapeutical strategy to improve clinical outcomes in patients with chronic kidney diseases. Int. J. Mol. Sci. 24 (10), 8732. 10.3390/ijms24108732 37240080 PMC10218404

[B15] Doherty M. Jenkins W. Richardson H. Sarmanova A. Abhishek A. Ashton D. (2018). Efficacy and cost-effectiveness of nurse-led care involving education and engagement of patients and a treat-to-target urate-lowering strategy versus usual care for gout: a randomised controlled trial. Lancet 392 (10156), 1403–1412. 10.1016/s0140-6736(18)32158-5 30343856 PMC6196879

[B16] Dong M. Chen H. Wen S. Yuan Y. Yang L. Xu D. (2023). The mechanism of sodium-glucose cotransporter-2 inhibitors in reducing uric acid in type 2 diabetes mellitus. Diabetes Metab. Syndr. Obes. 16, 437–445. 10.2147/dmso.s399343 36820272 PMC9938669

[B17] Doria A. Galecki A. T. Spino C. Pop-Busui R. Cherney D. Z. Lingvay I. (2020). Serum urate lowering with allopurinol and kidney function in type 1 diabetes. N. Engl. J. Med. 382 (26), 2493–2503. 10.1056/NEJMoa1916624 32579810 PMC7375708

[B18] Eriksson J. W. Lundkvist P. Jansson P. A. Johansson L. Kvarnström M. Moris L. (2018). Effects of dapagliflozin and n-3 carboxylic acids on non-alcoholic fatty liver disease in people with type 2 diabetes: a double-blind randomised placebo-controlled study. Diabetologia 61 (9), 1923–1934. 10.1007/s00125-018-4675-2 29971527 PMC6096619

[B19] Fathallah-Shaykh S. A. Cramer M. T. (2014). Uric acid and the kidney. Pediatr. Nephrol. 29 (6), 999–1008. 10.1007/s00467-013-2549-x 23824181

[B20] Fayazi H. S. Yaseri M. Mortazavi S. S. Sharifhassan Z. Assadinia A. S. (2022). The relation between serum uric acid levels and diabetic peripheral neuropathy in type 2 diabetes in Guilan, north of Iran. BMC Endocr. Disord. 22 (1), 39. 10.1186/s12902-022-00952-5 35151299 PMC8840027

[B21] Fernandes A. D. (2024). SAVE-care (sodium glucose cotransporter-2 inhibitors [SGLT2i] as novel gout care) trial (SAVE-Care). Available online at: https://clinicaltrials.gov/study/NCT06674109?cond=Gout&intr=SGLT2%20inhibitor&rank=3 (Accessed April 26, 2025).

[B22] Ferrannini E. Seman L. Seewaldt-Becker E. Hantel S. Pinnetti S. Woerle H. J. (2013). A Phase IIb, randomized, placebo-controlled study of the SGLT2 inhibitor empagliflozin in patients with type 2 diabetes. Diabetes Obes. Metab. 15 (8), 721–728. 10.1111/dom.12081 23398530

[B23] Ferreira J. P. Inzucchi S. E. Mattheus M. Meinicke T. Steubl D. Wanner C. (2022). Empagliflozin and uric acid metabolism in diabetes: a *post hoc* analysis of the EMPA-REG OUTCOME trial. Diabetes Obes. Metab. 24 (1), 135–141. 10.1111/dom.14559 34558768 PMC9293326

[B24] Fitchett D. (2019). A safety update on sodium glucose co-transporter 2 inhibitors. Diabetes Obes. Metab. 21 (Suppl. 2), 34–42. 10.1111/dom.13611 31081590

[B25] Gorgojo-Martínez J. J. Górriz J. L. Cebrián-Cuenca A. Castro Conde A. Velasco A. M. (2024). Clinical Recommendations for managing genitourinary adverse effects in patients treated with SGLT-2 inhibitors: a multidisciplinary expert consensus. J. Clin. Med. 13 (21), 6509. 10.3390/jcm13216509 39518647 PMC11546491

[B26] Group KDIGOKCW (2024). KDIGO 2024 clinical practice guideline for the evaluation and management of chronic kidney disease. Kidney Int. 105 (4s), S117–s314. 10.1016/j.kint.2023.10.018 38490803

[B27] Halvorsen Y. D. Conery A. L. Lock J. P. Zhou W. Freeman M. W. (2023). Bexagliflozin as an adjunct to metformin for the treatment of type 2 diabetes in adults: a 24-week, randomized, double-blind, placebo-controlled trial. Diabetes Obes. Metab. 25 (10), 2954–2962. 10.1111/dom.15192 37409573

[B28] Hao Z. Huang X. Shao H. Tian F. (2018). Effects of dapagliflozin on serum uric acid levels in hospitalized type 2 diabetic patients with inadequate glycemic control: a randomized controlled trial. Ther. Clin. Risk Manag. 14, 2407–2413. 10.2147/tcrm.s186347 30587997 PMC6294165

[B29] Häring H.-U. Merker L. Seewaldt-Becker E. Weimer M. Meinicke T. Woerle H. J. (2013). Empagliflozin as add-on to metformin plus sulfonylurea in patients with type 2 diabetes: a 24-week, randomized, double-blind, placebo-controlled trial. Diabetes Care 36 (11), 3396–3404. 10.2337/dc12-2673 23963895 PMC3816918

[B30] Heidenreich P. A. Bozkurt B. Aguilar D. Allen L. A. Byun J. J. Colvin M. M. (2022). 2022 AHA/ACC/HFSA guideline for the management of heart failure: a report of the American college of cardiology/American heart association joint committee on clinical practice guidelines. Circulation 145 (18), e895–e1032. 10.1161/cir.0000000000001063 35363499

[B31] Higgins J. P. T. Thomas J. Chandler J. Cumpston M. Li T. Page M. J. (2024). Cochrane handbook for systematic reviews of interventions (Cochrane), version 6.5.0. Available online at: https://training.cochrane.org/handbook/current (Accessed April 26, 2025). 10.1002/14651858.ED000142 PMC10284251 31643080

[B32] Hou Y. C. Zheng C. M. Yen T. H. Lu K. C. (2020). Molecular mechanisms of SGLT2 inhibitor on cardiorenal protection. Int. J. Mol. Sci. 21 (21), 7833. 10.3390/ijms21217833 33105763 PMC7660105

[B33] Hu X. Yang Y. Hu X. Jia X. Liu H. Wei M. (2022). Effects of sodium-glucose cotransporter 2 inhibitors on serum uric acid in patients with type 2 diabetes mellitus: a systematic review and network meta-analysis. Diabetes Obes. Metab. 24 (2), 228–238. 10.1111/dom.14570 34617381

[B34] Ito H. Abe M. Mifune M. Oshikiri K. Antoku S. Takeuchi Y. (2011). Hyperuricemia is independently associated with coronary heart disease and renal dysfunction in patients with type 2 diabetes mellitus. PLoS One 6 (11), e27817. 10.1371/journal.pone.0027817 22125626 PMC3220675

[B35] Ji L. Ma J. Li H. Mansfield T. A. T'Joen C. L. Iqbal N. (2014). Dapagliflozin as monotherapy in drug-naive Asian patients with type 2 diabetes mellitus: a randomized, blinded, prospective phase III study. Clin. Ther. 36 (1), 84–100.e9. 10.1016/j.clinthera.2013.11.002 24378206

[B36] Jiang H. Su Y. Liu R. Xu X. Xu Q. Yang J. (2025). Hyperuricemia and the risk of stroke incidence and mortality: a systematic review and meta-analysis. Arch. Rheumatol. 40 (1), 128–143. 10.46497/ArchRheumatol.2025.10808 40264487 PMC12010261

[B37] Kadowaki T. Haneda M. Inagaki N. Terauchi Y. Taniguchi A. Koiwai K. (2014). Empagliflozin monotherapy in Japanese patients with type 2 diabetes mellitus: a randomized, 12-week, double-blind, placebo-controlled, phase II trial. Adv. Ther. 31 (6), 621–638. 10.1007/s12325-014-0126-8 24958326

[B38] Kaku K. Watada H. Iwamoto Y. Utsunomiya K. Terauchi Y. Tobe K. (2014). Efficacy and safety of monotherapy with the novel sodium/glucose cotransporter-2 inhibitor tofogliflozin in Japanese patients with type 2 diabetes mellitus: a combined Phase 2 and 3 randomized, placebo-controlled, double-blind, parallel-group comparative study. Cardiovasc Diabetol. 13, 65. 10.1186/1475-2840-13-65 24678906 PMC4021346

[B39] Kario K. Okada K. Kato M. Nishizawa M. Yoshida T. Asano T. (2019). Twenty-four-hour blood pressure-lowering effect of a sodium-glucose cotransporter 2 inhibitor in patients with diabetes and uncontrolled nocturnal hypertension: results from the randomized, placebo-controlled SACRA study. Circulation 139 (18), 2089–2097. 10.1161/circulationaha.118.037076 30586745 PMC6493695

[B40] Kashiwagi A. Kazuta K. Takinami Y. Yoshida S. Utsuno A. Nagase I. (2015). Ipragliflozin improves glycemic control in Japanese patients with type 2 diabetes mellitus: the BRIGHTEN study. Diabetol. Int. 6 (1), 8–18. 10.1007/s13340-014-0164-0

[B41] Kashiwagi A. Kazuta K. Yoshida S. Nagase I. (2014). Randomized, placebo-controlled, double-blind glycemic control trial of novel sodium-dependent glucose cotransporter 2 inhibitor ipragliflozin in Japanese patients with type 2 diabetes mellitus. J. Diabetes Investig. 5 (4), 382–391. 10.1111/jdi.12156 PMC4210070 25411597

[B42] Katsiki N. Papanas N. Fonseca V. A. Maltezos E. Mikhailidis D. P. (2013). Uric acid and diabetes: is there a link? Curr. Pharm. Des. 19 (27), 4930–4937. 10.2174/1381612811319270016 23278493

[B43] Kochanowska A. Rusztyn P. Szczerkowska K. Surma S. Gąsecka A. Jaguszewski M. J. (2023). Sodium-glucose cotransporter 2 inhibitors to decrease the uric acid concentration-A novel mechanism of action. J. Cardiovasc Dev. Dis. 10 (7), 268. 10.3390/jcdd10070268 37504524 PMC10380892

[B44] Kodama S. Saito K. Yachi Y. Asumi M. Sugawara A. Totsuka K. (2009). Association between serum uric acid and development of type 2 diabetes. Diabetes Care 32 (9), 1737–1742. 10.2337/dc09-0288 19549729 PMC2732137

[B45] Kohan D. E. Fioretto P. Tang W. List J. F. (2014). Long-term study of patients with type 2 diabetes and moderate renal impairment shows that dapagliflozin reduces weight and blood pressure but does not improve glycemic control. Kidney Int. 85 (4), 962–971. 10.1038/ki.2013.356 24067431 PMC3973038

[B46] Kondo T. Butt J. H. Curtain J. P. Jhund P. S. Docherty K. F. Claggett B. L. (2023). Efficacy of dapagliflozin according to heart rate: a patient-level pooled analysis of DAPA-HF and deliver. Circ. Heart Fail 16 (12), e010898. 10.1161/circheartfailure.123.010898 37886880

[B47] Kovacs C. S. Seshiah V. Merker L. Christiansen A. V. Roux F. Salsali A. (2015). Empagliflozin as add-on therapy to pioglitazone with or without metformin in patients with type 2 diabetes mellitus. Clin. Ther. 37 (8), 1773–88.e1. 10.1016/j.clinthera.2015.05.511 26138864

[B48] Lee M. M. Y. Brooksbank K. J. M. Wetherall K. Mangion K. Roditi G. Campbell R. T. (2021a). Effect of empagliflozin on left ventricular volumes in patients with type 2 diabetes, or prediabetes, and heart failure with reduced ejection fraction (SUGAR-DM-HF). Circulation 143 (6), 516–525. 10.1161/circulationaha.120.052186 33186500 PMC7864599

[B49] Lee S. H. Min K. W. Lee B. W. Jeong I. K. Yoo S. J. Kwon H. S. (2021b). Effect of dapagliflozin as an add-on therapy to insulin on the glycemic variability in subjects with type 2 diabetes mellitus (dive): a multicenter, placebo-controlled, double-blind, randomized study. Diabetes Metab. J. 45 (3), 339–348. 10.4093/dmj.2019.0203 32602273 PMC8164951

[B50] Li B. Chen L. Hu X. Tan T. Yang J. Bao W. (2023a). Association of serum uric acid with all-cause and cardiovascular mortality in diabetes. Diabetes Care 46 (2), 425–433. 10.2337/dc22-1339 36490263

[B51] Li C. X. Liu L. Y. Zhang C. X. Geng X. H. Gu S. M. Wang Y. Q. (2023b). Comparative safety of different sodium-glucose transporter 2 inhibitors in patients with type 2 diabetes: a systematic review and network meta-analysis of randomized controlled trials. Front. Endocrinol. (Lausanne) 14, 1238399. 10.3389/fendo.2023.1238399 37701900 PMC10494439

[B52] Li J. Woodward M. Perkovic V. Figtree G. A. Heerspink H. J. L. Mahaffey K. W. (2020). Mediators of the effects of canagliflozin on heart failure in patients with type 2 diabetes. JACC Heart Fail 8 (1), 57–66. 10.1016/j.jchf.2019.08.004 31676303

[B53] Li M. Zhang J. Yang G. Zhang J. Han M. Zhang Y. (2023c). Effects of sodium-glucose cotransporter 2 inhibitors on renal risk factors in patients with abnormal glucose metabolism: a meta-analysis of randomized controlled trials. Eur. J. Clin. Pharmacol. 79 (6), 859–871. 10.1007/s00228-023-03490-8 37097298

[B54] Li X. Huang B. Liu Y. Wang M. Cui J. Q. (2025). Uric acid in diabetic microvascular complications: mechanisms and therapy. J. Diabetes Complicat. 39 (2), 108929. 10.1016/j.jdiacomp.2024.108929 39689504

[B55] Lv J. Guo L. Wang R. Chen J. (2023). Efficacy and safety of sodium-glucose cotransporter-2 inhibitors in nondiabetic patients with chronic kidney disease: a review of recent evidence. Kidney Dis. (Basel) 9 (5), 326–341. 10.1159/000530395 37901712 PMC10601939

[B56] Lv Q. Meng X. F. He F. F. Chen S. Su H. Xiong J. (2013). High serum uric acid and increased risk of type 2 diabetes: a systemic review and meta-analysis of prospective cohort studies. PLoS One 8 (2), e56864. 10.1371/journal.pone.0056864 23437258 PMC3577701

[B57] Lytvyn Y. Škrtić M. Yang G. K. Yip P. M. Perkins B. A. Cherney D. Z. (2015). Glycosuria-mediated urinary uric acid excretion in patients with uncomplicated type 1 diabetes mellitus. Am. J. Physiol. Ren. Physiol. 308 (2), F77–F83. 10.1152/ajprenal.00555.2014 25377916

[B58] Mackenzie I. S. Ford I. Nuki G. Hallas J. Hawkey C. J. Webster J. (2020). Long-term cardiovascular safety of febuxostat compared with allopurinol in patients with gout (FAST): a multicentre, prospective, randomised, open-label, non-inferiority trial. Lancet 396 (10264), 1745–1757. 10.1016/s0140-6736(20)32234-0 33181081

[B59] Matharu K. Chana K. Ferro C. J. Jones A. M. (2021). Polypharmacology of clinical sodium glucose co-transport protein 2 inhibitors and relationship to suspected adverse drug reactions. Pharmacol. Res. Perspect. 9 (5), e00867. 10.1002/prp2.867 34586753 PMC8480305

[B60] McDonagh T. A. Metra M. Adamo M. Gardner R. S. Baumbach A. Böhm M. (2023). 2023 Focused Update of the 2021 ESC Guidelines for the diagnosis and treatment of acute and chronic heart failure. Eur. Heart J. 44 (37), 3627–3639. 10.1093/eurheartj/ehad195 37622666

[B61] McDowell K. Welsh P. Docherty K. F. Morrow D. A. Jhund P. S. de Boer R. A. (2022). Dapagliflozin reduces uric acid concentration, an independent predictor of adverse outcomes in DAPA-HF. Eur. J. Heart Fail 24 (6), 1066–1076. 10.1002/ejhf.2433 35064721 PMC9540869

[B62] Mende C. (2015). Management of chronic kidney disease: the relationship between serum uric acid and development of nephropathy. Adv. Ther. 32 (12), 1177–1191. 10.1007/s12325-015-0272-7 26650815 PMC4679778

[B63] Mentz R. J. Brunton S. A. Rangaswami J. (2023). Sodium-glucose cotransporter-2 inhibition for heart failure with preserved ejection fraction and chronic kidney disease with or without type 2 diabetes mellitus: a narrative review. Cardiovasc Diabetol. 22 (1), 316. 10.1186/s12933-023-02023-y 37974185 PMC10655322

[B64] Mozawa K. Kubota Y. Hoshika Y. Tara S. Tokita Y. Yodogawa K. (2021). Empagliflozin confers reno-protection in acute myocardial infarction and type 2 diabetes mellitus. Esc. Heart Fail 8 (5), 4161–4173. 10.1002/ehf2.13509 34235875 PMC8497324

[B65] Novikov A. Fu Y. Huang W. Freeman B. Patel R. van Ginkel C. (2019). SGLT2 inhibition and renal urate excretion: role of luminal glucose, GLUT9, and URAT1. Am. J. Physiol. Ren. Physiol. 316 (1), F173–F185. 10.1152/ajprenal.00462.2018 PMC6383194 30427222

[B66] Otani N. Ouchi M. Kudo H. Tsuruoka S. Hisatome I. Anzai N. (2020). Recent approaches to gout drug discovery: an update. Expert Opin. Drug Discov. 15 (8), 943–954. 10.1080/17460441.2020.1755251 32329387

[B67] Packer M. (2024). Hyperuricemia and gout reduction by SGLT2 inhibitors in diabetes and heart failure: JACC review topic of the week. J. Am. Coll. Cardiol. 83 (2), 371–381. 10.1016/j.jacc.2023.10.030 38199714

[B68] Pan J. Yang Q. Peng J. Li A. Liu Y. Yi B. (2023). A cohort study on the correlation between serum uric acid trajectory and the progression of renal function in patients with Type 2 diabetes mellitus. Zhong Nan Da Xue Xue Bao Yi Xue Ban. 48 (5), 725–732. 10.11817/j.issn.1672-7347.2023.220539 37539575 PMC10930409

[B69] Pollock C. Stefánsson B. Reyner D. Rossing P. Sjöström C. D. Wheeler D. C. (2019). Albuminuria-lowering effect of dapagliflozin alone and in combination with saxagliptin and effect of dapagliflozin and saxagliptin on glycaemic control in patients with type 2 diabetes and chronic kidney disease (DELIGHT): a randomised, double-blind, placebo-controlled trial. Lancet Diabetes Endocrinol. 7 (6), 429–441. 10.1016/s2213-8587(19)30086-5 30992195

[B70] Qiu R. Capuano G. Meininger G. (2014). Efficacy and safety of twice-daily treatment with canagliflozin, a sodium glucose co-transporter 2 inhibitor, added on to metformin monotherapy in patients with type 2 diabetes mellitus. J. Clin. Transl. Endocrinol. 1 (2), 54–60. 10.1016/j.jcte.2014.04.001 29159083 PMC5685027

[B71] Ramírez-Rodríguez A. M. González-Ortiz M. Martínez-Abundis E. (2020). Effect of dapagliflozin on insulin secretion and insulin sensitivity in patients with prediabetes. Exp. Clin. Endocrinol. Diabetes 128 (8), 506–511. 10.1055/a-0664-7583 30149417

[B72] Refardt J. Imber C. Sailer C. O. Jeanloz N. Potasso L. Kutz A. (2020). A randomized trial of empagliflozin to increase plasma sodium levels in patients with the syndrome of inappropriate antidiuresis. J. Am. Soc. Nephrol. 31 (3), 615–624. 10.1681/asn.2019090944 32019783 PMC7062212

[B73] Rivera-De-la-Parra D. Hernández-Jiménez S. Almeda-Valdés P. Aguilar-Salinas C. A. Graue-Hernández E. O. Pérez-Peralta L. (2024). Association between uric acid and referable diabetic retinopathy in patients with type 2 diabetes. Sci. Rep. 14 (1), 12968. 10.1038/s41598-024-63340-0 38839883 PMC11153536

[B74] Roden M. Weng J. Eilbracht J. Delafont B. Kim G. Woerle H. J. (2013). Empagliflozin monotherapy with sitagliptin as an active comparator in patients with type 2 diabetes: a randomised, double-blind, placebo-controlled, phase 3 trial. Lancet Diabetes Endocrinol. 1 (3), 208–219. 10.1016/s2213-8587(13)70084-6 24622369

[B75] Rosenstock J. Aggarwal N. Polidori D. Zhao Y. Arbit D. Usiskin K. (2012a). Dose-ranging effects of canagliflozin, a sodium-glucose cotransporter 2 inhibitor, as add-on to metformin in subjects with type 2 diabetes. Diabetes Care 35 (6), 1232–1238. 10.2337/dc11-1926 22492586 PMC3357223

[B76] Rosenstock J. Vico M. Wei L. Salsali A. List J. F. (2012b). Effects of dapagliflozin, an SGLT2 inhibitor, on HbA1c, body weight, and hypoglycemia risk in patients with type 2 diabetes inadequately controlled on pioglitazone monotherapy. Diabetes Care 35 (7), 1473–1478. 10.2337/dc11-1693 22446170 PMC3379599

[B77] Ross S. Thamer C. Cescutti J. Meinicke T. Woerle H. J. Broedl U. C. (2015). Efficacy and safety of empagliflozin twice daily versus once daily in patients with type 2 diabetes inadequately controlled on metformin: a 16-week, randomized, placebo-controlled trial. Diabetes Obes. Metab. 17 (7), 699–702. 10.1111/dom.12469 25827441

[B78] Saha A. Samadder A. Nandi S. (2023). Stem cell therapy in combination with naturopathy: current progressive management of diabetes and associated complications. Curr. Top. Med. Chem. 23 (8), 649–689. 10.2174/1568026623666221201150933 36464871

[B79] Sant'Ana P. G. Tomasi L. C. Murata G. M. Vileigas D. F. Mota G. A. F. Souza S. L. B. (2023). Hypoxia-inducible factor 1-alpha and glucose metabolism during cardiac remodeling progression from hypertrophy to heart failure. Int. J. Mol. Sci. 24 (7), 6201. 10.3390/ijms24076201 37047174 PMC10094437

[B80] Sargeant J. A. Henson J. King J. A. Yates T. Khunti K. Davies M. J. (2019). A review of the effects of glucagon-like peptide-1 receptor agonists and sodium-glucose cotransporter 2 inhibitors on lean body mass in humans. Endocrinol. Metab. Seoul. 34 (3), 247–262. 10.3803/EnM.2019.34.3.247 31565876 PMC6769337

[B81] Scheen A. J. (2015). Pharmacodynamics, efficacy and safety of sodium-glucose co-transporter type 2 (SGLT2) inhibitors for the treatment of type 2 diabetes mellitus. Drugs 75 (1), 33–59. 10.1007/s40265-014-0337-y 25488697

[B82] Schumm-Draeger P. M. Burgess L. Korányi L. Hruba V. Hamer-Maansson J. E. de Bruin T. W. (2015). Twice-daily dapagliflozin co-administered with metformin in type 2 diabetes: a 16-week randomized, placebo-controlled clinical trial. Diabetes Obes. Metab. 17 (1), 42–51. 10.1111/dom.12387 25200570

[B83] Seino Y. Sasaki T. Fukatsu A. Imazeki H. Ochiai H. Sakai S. (2018). Efficacy and safety of luseogliflozin added to insulin therapy in Japanese patients with type 2 diabetes: a multicenter, 52-week, clinical study with a 16-week, double-blind period and a 36-week, open-label period. Curr. Med. Res. Opin. 34 (6), 981–994. 10.1080/03007995.2018.1441816 29448833

[B84] Seino Y. Sasaki T. Fukatsu A. Sakai S. Samukawa Y. (2014a). Efficacy and safety of luseogliflozin monotherapy in Japanese patients with type 2 diabetes mellitus: a 12-week, randomized, placebo-controlled, phase II study. Curr. Med. Res. Opin. 30 (7), 1219–1230. 10.1185/03007995.2014.901943 24597840

[B85] Seino Y. Sasaki T. Fukatsu A. Ubukata M. Sakai S. Samukawa Y. (2014b). Dose-finding study of luseogliflozin in Japanese patients with type 2 diabetes mellitus: a 12-week, randomized, double-blind, placebo-controlled, phase II study. Curr. Med. Res. Opin. 30 (7), 1231–1244. 10.1185/03007995.2014.909390 24673496

[B86] Seino Y. Sasaki T. Fukatsu A. Ubukata M. Sakai S. Samukawa Y. (2014c). Efficacy and safety of luseogliflozin as monotherapy in Japanese patients with type 2 diabetes mellitus: a randomized, double-blind, placebo-controlled, phase 3 study. Curr. Med. Res. Opin. 30 (7), 1245–1255. 10.1185/03007995.2014.912983 24708292

[B87] Shamseer L. Moher D. Clarke M. Ghersi D. Liberati A. Petticrew M. (2015). Preferred reporting items for systematic review and meta-analysis protocols (PRISMA-P) 2015: elaboration and explanation. Bmj 350, g7647. 10.1136/bmj.g7647 25555855

[B88] Shen Z. Xu L. Wu T. Wang H. Wang Q. Ge X. (2024). Structural basis for urate recognition and apigenin inhibition of human GLUT9. Nat. Commun. 15 (1), 5039. 10.1038/s41467-024-49420-9 38866775 PMC11169512

[B89] Søfteland E. Meier J. J. Vangen B. Toorawa R. Maldonado-Lutomirsky M. Broedl U. C. (2017). Empagliflozin as add-on therapy in patients with type 2 diabetes inadequately controlled with linagliptin and metformin: a 24-week randomized, double-blind, parallel-group trial. Diabetes Care 40 (2), 201–209. 10.2337/dc16-1347 27913576

[B90] Sridhar V. S. Heerspink H. J. L. Davies M. J. Banks P. Girard M. Garg S. K. (2023). The effects of sotagliflozin in type 1 diabetes on clinical markers associated with cardiorenal protection: an exploratory analysis of inTandem3. Diabetes Care 46 (7), e133–e135. 10.2337/dc23-0129 37172207 PMC10300514

[B91] Sridharan K. Alkhidir M. (2025). Hypouricemic effect of sodium glucose transporter-2 inhibitors: a network meta-analysis and meta-regression of randomized clinical trials. Expert Rev. Endocrinol. Metab. 20 (2), 139–146. 10.1080/17446651.2025.2456504 39835962

[B92] Stack A. G. Han D. Goldwater R. Johansson S. Dronamraju N. Oscarsson J. (2021). Dapagliflozin added to verinurad plus febuxostat further reduces serum uric acid in hyperuricemia: the QUARTZ study. J. Clin. Endocrinol. Metab. 106 (5), e2347–e2356. 10.1210/clinem/dgaa748 33075806 PMC8063233

[B93] Stenlöf K. Cefalu W. T. Kim K. A. Alba M. Usiskin K. Tong C. (2013). Efficacy and safety of canagliflozin monotherapy in subjects with type 2 diabetes mellitus inadequately controlled with diet and exercise. Diabetes Obes. Metab. 15 (4), 372–382. 10.1111/dom.12054 23279307 PMC3593184

[B94] Strojek K. Yoon K. H. Hruba V. Elze M. Langkilde A. M. Parikh S. (2011). Effect of dapagliflozin in patients with type 2 diabetes who have inadequate glycaemic control with glimepiride: a randomized, 24-week, double-blind, placebo-controlled trial. Diabetes Obes. Metab. 13 (10), 928–938. 10.1111/j.1463-1326.2011.01434.x 21672123

[B95] Suijk D. L. S. van Baar M. J. B. van Bommel E. J. M. Iqbal Z. Krebber M. M. Vallon V. (2022). SGLT2 inhibition and uric acid excretion in patients with type 2 diabetes and normal kidney function. Clin. J. Am. Soc. Nephrol. 17 (5), 663–671. 10.2215/cjn.11480821 35322793 PMC9269569

[B96] Tanaka M. Yamakage H. Inoue T. Odori S. Kusakabe T. Shimatsu A. (2020). Beneficial effects of ipragliflozin on the renal function and serum uric acid levels in Japanese patients with type 2 diabetes: a randomized, 12-week, open-label, active-controlled trial. Intern Med. 59 (5), 601–609. 10.2169/internalmedicine.3473-19 32115517 PMC7086326

[B97] Tao H. Mo Y. Liu W. Wang H. (2023). A review on gout: looking back and looking ahead. Int. Immunopharmacol. 117, 109977. 10.1016/j.intimp.2023.109977 37012869

[B98] Terauchi Y. Tamura M. Senda M. Gunji R. Kaku K. (2017). Efficacy and safety of tofogliflozin in Japanese patients with type 2 diabetes mellitus with inadequate glycaemic control on insulin therapy (J-STEP/INS): results of a 16-week randomized, double-blind, placebo-controlled multicentre trial. Diabetes Obes. Metab. 19 (10), 1397–1407. 10.1111/dom.12957 28371205 PMC5637911

[B99] Tikkanen I. Narko K. Zeller C. Green A. Salsali A. Broedl U. C. (2015). Empagliflozin reduces blood pressure in patients with type 2 diabetes and hypertension. Diabetes Care 38 (3), 420–428. 10.2337/dc14-1096 25271206

[B100] Tseng C. H. (2004). Independent association of uric acid levels with peripheral arterial disease in Taiwanese patients with Type 2 diabetes. Diabet. Med. 21 (7), 724–729. 10.1111/j.1464-5491.2004.01239.x 15209765

[B101] U.S. FOOD and DRUG ADMINISTRATION (2019). FDA adds Boxed Warning for increased risk of death with gout medicine Uloric (febuxostat). Available online at: https://www.fda.gov/drugs/drug-safety-and-availability/fda-adds-boxed-warning-increased-risk-death-gout-medicine-uloric-febuxostat#:∼:text=This%20is%20an%20update%20to%20the%20FDA%20Drug,medicine%20febuxostat%20%28Uloric%29%20issued%20on%20November%2015%2C%202017 (Accessed April 20, 2025).

[B102] U.S. FOOD and DRUG ADMINISTRATION (2021). Sodium-glucose cotransporter-2 (SGLT2) inhibitors. Available online at: https://www.fda.gov/drugs/drugsafety/postmarketdrugsafetyinformationforpatientsandproviders/ucm446852.htm (Accessed October 5, 2024).

[B103] Vallon V. (2024). State-of-the-Art-Review: mechanisms of action of SGLT2 inhibitors and clinical implications. Am. J. Hypertens. 37 (11), 841–852. 10.1093/ajh/hpae092 39017631 PMC11471837

[B104] van Raalte D. H. Bjornstad P. Persson F. Powell D. R. de C. C. R. Wang P. S. (2019). The impact of sotagliflozin on renal function, albuminuria, blood pressure, and hematocrit in adults with type 1 diabetes. Diabetes Care 42 (10), 1921–1929. 10.2337/dc19-0937 31371432 PMC6905482

[B105] Verma S. Dhingra N. K. Butler J. Anker S. D. Ferreira J. P. Filippatos G. (2022). Empagliflozin in the treatment of heart failure with reduced ejection fraction in addition to background therapies and therapeutic combinations (EMPEROR-Reduced): a post-hoc analysis of a randomised, double-blind trial. Lancet Diabetes Endocrinol. 10 (1), 35–45. 10.1016/s2213-8587(21)00292-8 34861154

[B106] Weber M. A. Mansfield T. A. Cain V. A. Iqbal N. Parikh S. Ptaszynska A. (2016b). Blood pressure and glycaemic effects of dapagliflozin versus placebo in patients with type 2 diabetes on combination antihypertensive therapy: a randomised, double-blind, placebo-controlled, phase 3 study. Lancet Diabetes Endocrinol. 4 (3), 211–220. 10.1016/s2213-8587(15)00417-9 26620248

[B107] Weber M. A. Mansfield T. A. Alessi F. Iqbal N. Parikh S. Ptaszynska A. (2016a). Effects of dapagliflozin on blood pressure in hypertensive diabetic patients on renin-angiotensin system blockade. Blood Press 25 (2), 93–103. 10.3109/08037051.2015.1116258 26623980

[B108] Wei J. Choi H. K. Dalbeth N. Li X. Li C. Zeng C. (2023). Gout flares and mortality after sodium-glucose cotransporter-2 inhibitor treatment for gout and type 2 diabetes. JAMA Netw. Open 6 (8), e2330885. 10.1001/jamanetworkopen.2023.30885 37624597 PMC10457713

[B109] Wilding J. P. Charpentier G. Hollander P. González-Gálvez G. Mathieu C. Vercruysse F. (2013a). Efficacy and safety of canagliflozin in patients with type 2 diabetes mellitus inadequately controlled with metformin and sulphonylurea: a randomised trial. Int. J. Clin. Pract. 67 (12), 1267–1282. 10.1111/ijcp.12322 24118688 PMC4282288

[B110] Wilding J. P. Ferrannini E. Fonseca V. A. Wilpshaar W. Dhanjal P. Houzer A. (2013b). Efficacy and safety of ipragliflozin in patients with type 2 diabetes inadequately controlled on metformin: a dose-finding study. Diabetes Obes. Metab. 15 (5), 403–409. 10.1111/dom.12038 23163880

[B111] Yang W. Ma J. Li Y. Li Y. Zhou Z. Kim J. H. (2018). Dapagliflozin as add-on therapy in Asian patients with type 2 diabetes inadequately controlled on insulin with or without oral antihyperglycemic drugs: a randomized controlled trial. J. Diabetes 10 (7), 589–599. 10.1111/1753-0407.12634 29215189

[B112] Yuan J. Zhao J. Qin Y. Xing Y. Yu Z. Zhang Y. (2024). Association of serum uric acid with all-cause and cardiovascular mortality in chronic kidney disease stages 3-5. Nutr. Metab. Cardiovasc Dis. 34 (6), 1518–1527. 10.1016/j.numecd.2024.01.032 38508991

[B113] Zanchi A. Pruijm M. Muller M. E. Ghajarzadeh-Wurzner A. Maillard M. Dufour N. (2022). Twenty-four hour blood pressure response to empagliflozin and its determinants in normotensive non-diabetic subjects. Front. Cardiovasc Med. 9, 854230. 10.3389/fcvm.2022.854230 35391843 PMC8981729

[B114] Zelniker T. A. Wiviott S. D. Raz I. Im K. Goodrich E. L. Bonaca M. P. (2019). SGLT2 inhibitors for primary and secondary prevention of cardiovascular and renal outcomes in type 2 diabetes: a systematic review and meta-analysis of cardiovascular outcome trials. Lancet 393 (10166), 31–39. 10.1016/s0140-6736(18)32590-x 30424892

[B115] Zhang P. Sun H. Cheng X. Li Y. Zhao Y. Mei W. (2022). Dietary intake of fructose increases purine *de novo* synthesis: a crucial mechanism for hyperuricemia. Front. Nutr. 9, 1045805. 10.3389/fnut.2022.1045805 36601078 PMC9807165

[B116] Zhao Y. Xu L. Tian D. Xia P. Zheng H. Wang L. (2018). Effects of sodium-glucose co-transporter 2 (SGLT2) inhibitors on serum uric acid level: a meta-analysis of randomized controlled trials. Diabetes Obes. Metab. 20 (2), 458–462. 10.1111/dom.13101 28846182

[B117] Zhu Y. Pandya B. J. Choi H. K. (2012). Comorbidities of gout and hyperuricemia in the US general population: NHANES 2007-2008. Am. J. Med. 125 (7), 679–87.e1. 10.1016/j.amjmed.2011.09.033 22626509

