# Supplementary material for: Serum uric acid reduction through SGLT2 inhibitors: evidence from a systematic review and meta-analysis
Source: Front Pharmacol. 2025 Jun 19;16:1551390. doi: 10.3389/fphar.2025.1551390 (PMC12223426; doi:10.3389/fphar.2025.1551390)
Supplement: Supplementary file 1 [file Table1.DOCX]

**Supplemental Table 1.** Risk of bias assessments of all included studies (n=51)

| **Author** | **D1** | **D2** | **D3** | **D4** | **D5** | **Overall** |
| --- | --- | --- | --- | --- | --- | --- |
| Li, 2020(47) | Low | Low | Low | Low | Low | Low |
| Ferreira, 2022(32) | Low | Low | Low | Low | Low | Low |
| Doehner, 2022(70) | Low | Low | Low | Low | Low | Low |
| Anker, 2021(26) | Low | Low | Low | Low | Low | Low |
| Butt, 2023a (43) | Low | Low | Some concerns | Low | Low | Some concerns |
| Strojek, 2011(65) | Low | Low | Low | Low | Low | Low |
| Rosenstock, 2012a (54) | Low | Low | Low | Low | Low | Low |
| Rosenstock, 2012b (55) | Low | Low | Some concerns | Low | Low | Some concerns |
| Bailey, 2013(27) | Low | Low | Low | Low | Low | Low |
| Bode, 2013(29) | Low | Low | Low | Low | Low | Low |
| Haring, 2013(35) | Low | Low | Low | Low | Low | Low |
| Roden, 2013(53) | Low | Low | Low | Low | Low | Low |
| Stenlöf, 2013(64) | Low | Low | Low | Low | Low | Low |
| Wilding, 2013a (73) | Low | Low | Low | Low | Low | Low |
| Wilding, 2013b (74) | Low | Low | Low | Low | Low | Low |
| Kadowaki, 2014(37) | Low | Low | Low | Low | Low | Low |
| Kashiwagi, 2014(41) | Low | Low | Low | Low | Low | Low |
| Qiu, 2014(50) | Low | Low | Low | Low | Low | Low |
| Barnett, 2014(28) | Low | Low | Low | Low | Low | Low |
| Bolinder, 2012(30) | Low | Low | Low | Low | Low | Low |
| Eriksson, 2018(31) | Low | Low | Some concerns | Low | Low | Some concerns |
| Ji, 2014(36) | Low | Low | Low | Low | Low | Low |
| Kaku, 2014(38) | Low | Low | Some concerns | Low | Low | Some concerns |
| Kario, 2018(39) | Low | Low | Low | Low | Low | Low |
| Kashiwagi, 2015(40) | Low | Low | Low | Low | Low | Low |
| Kohan, 2014(42) | Low | Low | Low | Low | Low | Low |
| Kovacs, 2015(44) | Low | Low | Low | Low | Low | Low |
| Lee, 2021a (45) | Low | Low | Some concerns | Low | Low | Some concerns |
| Lee, 2021b (46) | Low | Low | Low | Low | Low | Low |
| Mozawa, 2021(48) | Low | Low | Low | Low | Low | Low |
| Pollock, 2019(49) | Low | Low | Low | Low | Low | Low |
| Ramírez- Rodríguez, 2020(51) | Low | Low | Some concerns | Low | Low | Some concerns |
| Refardt, 2020(52) | Low | Low | Low | Low | Low | Low |
| Ross, 2015(56) | Low | Low | Low | Low | Low | Low |
| Schumm-Draeger, 2014(57) | Low | Low | Low | Low | Low | Low |
| Seino, 2014a (59) | Low | Low | Low | Low | Low | Low |
| Seino, 2014b (60) | Low | Low | Low | Low | Low | Low |
| Seino, 2014c (61) | Low | Low | Low | Low | Low | Low |
| Seino, 2018(58) | Low | Low | Low | Low | Low | Low |
| Søfteland, 2017(62) | Low | Low | Low | Low | Low | Low |
| Terauchi, 2017(67) | Low | Low | Low | Low | Low | Low |
| Tikkanen, 2015(68) | Low | Low | Low | Low | Low | Low |
| Van Raalte, 2019(69) | Low | Low | Some concerns | Low | Low | Some concerns |
| Weber, 2016a (72) | Low | Low | Low | Low | Low | Low |
| Weber, 2016b (71) | Low | Low | Low | Low | Low | Low |
| Yang, 2018(75) | Low | Low | Low | Low | Low | Low |
| Zanchi, 2022(76) | Low | Low | Low | Low | Low | Low |
| Hao, 2018(34) | Low | Low | Low | Low | Low | Low |
| Tanaka, 2020(66) | Low | Low | Low | Low | Low | Low |
| Halvorsen,2023(33) | Low | Low | Low | Low | Low | Low |
| Sridhar,2023(63) | Low | Low | Some concerns | Low | Low | Some concerns |

**Footnotes:**

D1: Risk of bias arising from the randomization process; D2: Risk of bias due to deviations from the intended interventions; D3: Risk of bias due to missing outcome data; D4: Risk of bias in measurement of the outcome; D5: Risk of bias in selection of the reported result; Overall: Overall risk of bias
