# Supplementary material for: Serum uric acid reduction through SGLT2 inhibitors: evidence from a systematic review and meta-analysis
Source: Front Pharmacol. 2025 Jun 19;16:1551390. doi: 10.3389/fphar.2025.1551390 (PMC12223426; doi:10.3389/fphar.2025.1551390)
Supplement: Supplementary file 2 [file Table2.docx]

**Supplementary Table 2. Subgroup analysis of primary outcome**

**SGLT2 inhibitors versus placebo**

| **Outcome or Subgroup** | **Studies** | **Participants** | **Statistical Method** | **Effect Estimate** |
| --- | --- | --- | --- | --- |
| **1.1 Canagliflozin** | 5 | 3370 | Risk Ratio (M-H, Fixed, 95% CI) | -22.86 [-30.87, -14.86] |
| **1.1.1 Canagliflozin 100mg** | 1 | 478 | Risk Ratio (M-H, Fixed, 95% CI) | -12.20 [-14.96, -9.44] |
| **1.1.2 Canagliflozin 300mg** | 1 | 475 | Risk Ratio (M-H, Fixed, 95% CI) | -13.20 [-16.02, -10.38] |
| **1.1.3 Canagliflozin 100mg** | 1 | 186 | Risk Ratio (M-H, Fixed, 95% CI) | -12.80 [-17.42, -8.18] |
| **1.1.4 Canagliflozin 300mg** | 1 | 186 | Risk Ratio (M-H, Fixed, 95% CI) | -11.00 [-17.63, -4.37] |
| **1.1.5 Canagliflozin 100mg** | 1 | 129 | Risk Ratio (M-H, Fixed, 95% CI) | -39.27[-41.45, -37.09] |
| **1.1.6 Canagliflozin 200mg** | 1 | 130 | Risk Ratio (M-H, Fixed, 95% CI) | -54.14[-56.55, -51.73] |
| **1.1.7 Canagliflozin 300mg** |  | 129 | Risk Ratio (M-H, Fixed, 95% CI) | -35.70[-37.77, -33.63] |
| **1.1.8 Canagliflozin 300mg bid** | 1 | 129 | Risk Ratio (M-H, Fixed, 95% CI) | -38.08 [-40.15, -36.01] |
| **1.1.9 Canagliflozin50mg** | 1 | 129 | Risk Ratio (M-H, Fixed, 95% CI) | -28.56[-30.86, -26.26] |
| **1.1.10 Canagliflozin 100mg** | 1 | 129 | Risk Ratio (M-H, Fixed, 95% CI) | -15.20[-18.59, -11.81] |
| **1.1.11 Canagliflozin 300mg** | 1 | 387 | Risk Ratio (M-H, Fixed, 95% CI) | -16.10[-19.44, -12.76] |
| **1.1.12 Canagliflozin 100mg** | 1 | 313 | Risk Ratio (M-H, Fixed, 95% CI) | -9.50[-14.00, -5.00] |
| **1.1.13 Canagliflozin 300mg** | 1 | 312 | Risk Ratio (M-H, Fixed, 95% CI) | -10.10[-14.29, -5.91] |
| **1.2 Dapagliflozin** | 16 | 5781 | Risk Ratio (M-H, Fixed, 95% CI) | -34.44 [-40.18, -28.70] |
| **1.3 Empagliflozin** | 17 | 22790 | Std. Mean Difference (IV, Fixed, 95% CI) | -45.61 [-52.26, 38.97] |
| **1.4 Ipragliflozin** | 3 | 1266 | Risk Ratio (M-H, Fixed, 95% CI) | -20.86 [-29.57, -12.15] |
| **1.5 Luseogliflozin** | 4 | 1186 | Risk Ratio (M-H, Fixed, 95% CI) | -24.53 [-34.32, -14.73] |
| **1.6 Sotagliflozin** | 2 | 3503 | Risk Ratio (M-H, Fixed, 95% CI) | -13.72 [-19.16, -8.29] |
| **1.7 Tofogliflozin** | 2 | 552 | Risk Ratio (M-H, Fixed, 95% CI) | -19.53 [-27.09, -11.98] |
| **1.8 Bexagliflozin** | 1 | 317 | Risk Ratio (M-H, Fixed, 95% CI) | -33.10[-45.14, -21.06] |

## T2DM versus other disease

| **Outcome or Subgroup** | **Studies** | **Participants** | **Statistical Method** | **Effect Estimate** |
| --- | --- | --- | --- | --- |
| **2.1 Type 2 diabetes mellitus** | 45 | 31120 | Risk Ratio (M-H, Random, 95% CI) | -21.90 [-22.01, -21.79] |
| **2.2 Type 1 diabetes mellitus** | 1 | 1402 | Risk Ratio (M-H, Random, 95% CI) | -14.60 [-19.50, -9.70] |
| **2.3 Partial diabetes** | 3 | 6180 | Std. Mean Difference (IV, Random, 95% CI) | -47.60 [-47.69, -47.51] |
| **2.4 Non-diabetes** | 2 | 63 | Std. Mean Difference (IV, Fixed, 95% CI) | -92.66 [-114.86, -70.45] |

**Footnotes:** CI=95% confidence intervals
